# Is there an increased risk of perinatal mental disorder in women with gestational diabetes? A systematic review and meta‐analysis

**DOI:** 10.1111/dme.14170

**Published:** 2019-11-29

**Authors:** C. A. Wilson, J. Newham, J. Rankin, K. Ismail, E. Simonoff, R. M. Reynolds, N. Stoll, L. M. Howard

**Affiliations:** ^1^ Section of Women's Mental Health King's College London and South London and Maudsley NHS Foundation Trust London UK; ^2^ Department of Psychological Medicine King's College London and South London and Maudsley NHS Foundation Trust London UK; ^3^ Department of Child and Adolescent Psychiatry King's College London and South London and Maudsley NHS Foundation Trust London UK; ^4^ Department of Primary Care and Public Health Sciences King's College London London UK; ^5^ Institute of Health and Society Newcastle University Newcastle upon Tyne UK; ^6^ Centre for Cardiovascular Science University of Edinburgh Edinburgh UK

## Abstract

**Aim:**

Gestational diabetes (GDM) and mental disorder are common perinatal morbidities and are associated with adverse maternal and child outcomes. While there is a relationship between type 2 diabetes and mental disorder, the relationship between GDM and mental disorder has been less studied. We conducted a systematic review and meta‐analysis of the prevalence of mental disorders in women with GDM and their risk for mental disorders compared with women without GDM.

**Methods:**

Published, peer‐reviewed literature measuring prevalence and/or odds of GDM and perinatal mental disorders was reviewed systematically. Risk of bias was assessed using a checklist. Two independent reviewers were involved. Analyses were grouped by stage of peripartum, i.e. antepartum at the time of GDM diagnosis and after diagnosis, and in the postpartum.

**Results:**

Sixty‐two studies were included. There was an increased risk of depressive symptoms in the antenatal period around the time of diagnosis of GDM [odds ratio (OR) 2.08; 95% confidence interval (CI) 1.42, 3.05] and in the postnatal period (OR 1.59; 95% CI 1.26, 2.00).

**Conclusions:**

Given the potential relationship between GDM and perinatal mental disorders, integration of physical and mental healthcare in women experiencing GDM and mental disorders could improve short‐ and long‐term outcomes for women and their children.

## What's new?

- Type 2 diabetes is associated with an increased risk of mental disorder, particularly depression.
- There is some emerging evidence that gestational diabetes (GDM) may also be associated with mental disorder, particularly postnatal depression.
- GDM is associated with an increased risk of both antenatal and postnatal depressive symptoms, with the highest risk around the time of GDM diagnosis.
- All healthcare professionals working with women with GDM should be aware of this increased risk for mental disorder because effective treatment of the disorder could improve outcomes for women and their children.

**What's new?**

- Type 2 diabetes is associated with an increased risk of mental disorder, particularly depression.
- There is some emerging evidence that gestational diabetes (GDM) may also be associated with mental disorder, particularly postnatal depression.
- GDM is associated with an increased risk of both antenatal and postnatal depressive symptoms, with the highest risk around the time of GDM diagnosis.
- All healthcare professionals working with women with GDM should be aware of this increased risk for mental disorder because effective treatment of the disorder could improve outcomes for women and their children.

## Introduction

Gestational diabetes (GDM) is defined as ‘glucose intolerance with onset during pregnancy’. Its global prevalence is between 5% and 10%, which varies depending on the diagnostic criteria employed and the population studied. The prevalence is increasing, mirroring general upward trends in non‐communicable disease and obesity prevalence. GDM is associated with adverse outcomes for mother and baby, including obstetric complications such as emergency Caesarean delivery and longer‐term risks of subsequent type 2 diabetes in mothers 1. In children, there may be increased risk of metabolic syndrome later in life 1 and adverse neuro‐behavioural outcomes, for example hyperactivity and lower verbal IQ scores 2.

Mental disorder is the most common morbidity of the peripartum (during pregnancy and up to 1 year following delivery), with one in five women developing a mental disorder during pregnancy or in the year following birth 3. It is also associated with adverse maternal and fetal outcomes, and emotional and behavioural problems in the child 4.

There is a growing body of literature suggesting a bidirectional relationship between type 2 diabetes and mental disorder, particularly depression. A range of mechanisms has been studied, such as inflammation and hypothalamic–pituitary–adrenal axis dysregulation, and shared socio‐environmental risk factors such as obesity and deprivation 5. Given that there is pathophysiology common to both GDM and type 2 diabetes, i.e. insulin resistance, there may be a hypothesized association between GDM and mental disorder.

As with the type 2 diabetes literature, most research on GDM and mental disorders to date has focused on depression, either in the postpartum (up to 1 year) following GDM or cross‐sectional associations in the antepartum 6, 7, 8. A recent review focused only on studies relating to postnatal depression 9.

The aim of this study was to conduct a systematic review and meta‐analysis of the prevalence of a wider range of mental disorders than investigated in previous reviews in women with GDM and their risk for subsequent mental disorder in the peripartum compared with women without GDM. Greater understanding of the risk for perinatal mental disorder in women with GDM could help to provide more tailored support to these women.

## Methods

The review followed Meta‐Analyses and Systematic Reviews of Observational Studies (MOOSE) 10 and Preferred Reporting Items for Systematic Reviews and Meta‐Analyses (PRISMA) guidelines 11. It was registered with PROSPERO (CRD42016041677).

### Data sources

Medline, PsycINFO, EMBASE and CINAHL were searched separately from inception until 25 April 2019. Cochrane Library and ClinicalTrials.gov were also searched using the same period. Search terms used for Medline, PsycINFO, EMBASE, CINAHL and Cochrane Library were adapted from previous systematic reviews in the area 12, 13 and Cochrane specialized registers 14, 15 (Appendix S1). Forward and backward citation tracking was also undertaken.

### Study selection

Inclusion criteria were: published, peer‐reviewed observational and intervention studies in any language, measuring GDM and perinatal mental disorder occurring in the same pregnancy. In intervention studies, only baseline measurements of mental health were eligible (as opposed to follow‐up data). Perinatal mental disorder was defined as antenatal (between conception and delivery) or postpartum (up to 1 year following delivery) mood, anxiety, psychotic or eating disorders, as there were plausible mechanisms for an association between these disorders and GDM. Mental disorder could be measured either by medical records or diagnostic and screening measures.

Exclusion criteria were: studies classifying mental disorder based solely on medication status due to the risk of misclassification bias when psychotropic prescriptions alone are used to identify mental disorder 16. Studies which did not provide data separately for antenatal and postnatal periods were ineligible, as hypothesized mechanisms of association, are potentially different for the antepartum and postpartum. Studies were also ineligible if mental disorder was known to have been measured prior to the onset of GDM: either pre‐pregnancy or in early pregnancy. In studies where mental disorder was clearly measured during pregnancy but there was uncertainty about when in pregnancy the mental disorder was measured, these studies were included in the review but excluded from meta‐analysis. In studies where there was uncertainty about pre‐gestational diabetes (type 1 and type 2 diabetes) being excluded from the control (non GDM) population, only prevalence data were used for meta‐analysis.

**Figure 1:**
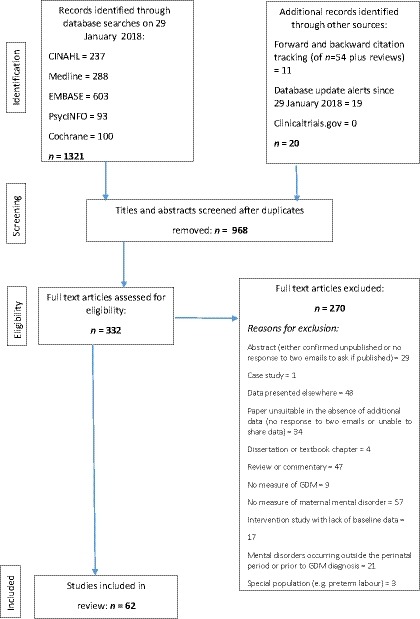
Flow diagram of study selection.

Following de‐duplication, titles and abstracts were screened, followed by full text screening by two independent reviewers. Sixty‐two studies met the inclusion criteria (Fig. 1) (see Appendix S2 for a list of the studies).

### Data extraction

Data extraction was conducted by two independent reviewers and included study characteristics such as study location, design and sample size, measurement of GDM and mental disorder, and inclusion and exclusion criteria. Prevalence and odds ratios (ORs) and any information on potential mechanisms were also extracted. Three of the included papers required translation to English. The authors of 41 studies were e‐mailed at the data extraction stage to request raw data or clarify an aspect of their methods. Following a second e‐mail reminder, replies were received from 17. Raw data were provided by four.

### Risk of bias assessment

A component approach to assessment of risk of bias was employed, as per current PRISMA guidelines 11. A modified Newcastle–Ottawa Scale 17 (piloted prior to use) was used (Table S1) by two independent reviewers. Of most interest were measurement and selection biases and the inclusion of significant confounders as most of the studies were anticipated to be of observational design and these sources of bias are most likely to impact on the results of an observational study. Each item was assigned a score from zero (high risk of bias) to two (low risk of bias). Selection bias was scored via an assessment of: (1) sample representativeness and (2) participation rates. Measurement bias was scored via an assessment of: (1) measure of GDM and (2) measure of mental disorder. A study with a score of zero in any of these four elements or on the element of inclusion of confounders in the design or analysis was deemed at high risk of bias. Otherwise studies were deemed at low to moderate risk.

### Data synthesis

**Figure 2:**
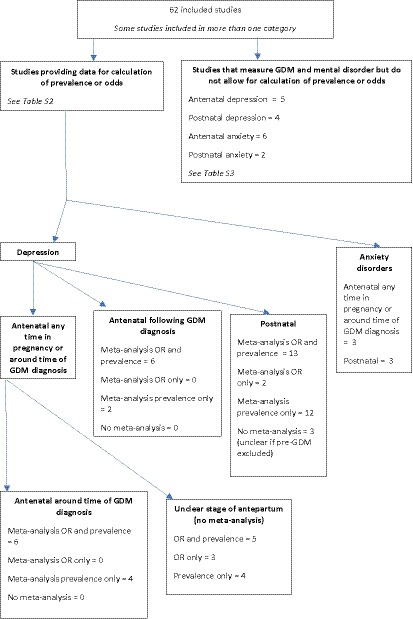
Flow diagram of how data from the 62 studies are presented.

Studies were grouped by mental disorder and timings of exposure, i.e. symptoms measured during the antepartum (cross‐sectionally at the time of GDM diagnosis and after diagnosis) and symptoms measured during the postpartum. Some papers presented only prevalence data. In such cases, ORs were calculated from this data (or raw data provided by authors). If ORs for at least five studies were available for each disorder at each period, meta‐analysis was undertaken 18. If there was any doubt as to whether or not pregestational diabetes had been excluded from the comparison group without GDM, the ORs for these studies were not included in the meta‐analysis. Figure 2 provides an overview of how the results of the 62 studies are presented.

Data were analysed using Stata 15. Metan and metaprop commands were used to produce pooled unadjusted ORs and prevalence and 95% confidence intervals (CIs) displayed as forest plots. If at least five adjusted ORs had been available, meta‐analysis would have been repeated using these estimates but this was not available. DerSimonian‐Laird random effects meta‐analysis 19 was used because there was expected to be a degree of heterogeneity between studies 20, 21. Heterogeneity was assessed using *I* ^2^, the proportion of total variation in study estimates that is due to heterogeneity 22. It was decided *a priori* that *I* ^2^ > 90% would preclude meta‐analysis as this represents considerable heterogeneity 23. Some of the prevalence meta‐analyses produced *I* ^2^ > 90%; in these circumstances prevalence is presented as median with interquartile range (IQR) as a standard summary measure of non‐parametric data. Sensitivity analyses on effect of risk of bias and screening tools vs. diagnostic codes as measures of mental disorder were conducted when sufficient studies were available. Cumulative meta‐analysis was used in a leave one out approach using the metacum command to investigate the impact of sample size on the final pooled effect estimate.

Publication bias was assessed using funnel plots for meta‐analyses with at least 10 studies using metafunnel command to assess association between study size and effect size 24. These were examined for evidence of asymmetry via visual inspection and Egger's test for small study effects (metabias command) 25.

## Results

### Study characteristics

An overview of study characteristics for all 62 included papers is provided in Table 1. Fuller descriptions of the study characteristics are available in Tables S2 and S3. Meta‐analyses were conducted only for studies measuring depression as there were insufficient studies for other mental disorders; narrative syntheses are presented for anxiety disorders, although some of the studies measured both depression and anxiety symptoms. Although other mental disorders such as psychotic and eating disorders were included in the search, only studies measuring anxiety and depression met selection criteria due to the exclusion of studies in which mental disorder started prior to the diagnosis of GDM.

**Table 1:** Characteristics of included studies

	Antenatal depression	Postnatal depression	Antenatal anxiety	Postnatal anxiety
Total number	34	34	9	5
Mental disorder measure *N* (%)				
Diagnoses	4 (12)	9 (26)	1 (11)	3 (60)
Screening tools (total)	29 (85)	22 (65)	8 (89)	1 (20)
EPDS	10	14	0	0
BDI	5	1	0	0
CES‐D	4	4	0	0
PHQ‐9	4	2	0	0
DASS	2	0	2	0
MHI‐5	1	0	0	0
MADRS	1	1	0	0
Kessler 6	1	0	0	0
Zung SDS	1	0	0	0
STAI	0	0	5	1
Taylor Anxiety	0	0	1	0
Self report	1 (3)	3 (9)	0 (0)	1 (20)
Sample size *N* (%)				
< 100	7 (21)	4 (12)	1 (11)	0 (0)
100–500	13 (38)	4 (12)	8 (89)	1 (20)
> 500	14 (41)	26 (76)	0 (0)	4 (80)
Study design *N* (%)				
Cross‐sectional	13 (38)	3 (9)	4 (44)	0 (0)
Case–control study	2 (6)	2 (6)	0 (0)	0 (0)
Intervention study	2 (6)	2 (6)	2 (23)	0 (0)
Prospective cohort	15 (44)	20 (59)	3 (33)	3 (60)
Retrospective cohort	2 (6)	7 (20)	0 (0)	2 (40)
Location *N* (%)				
Africa	1 (3)	0 (0)	0 (0)	0 (0)
Asia	9 (26)	8 (24)	3 (33)	0 (0)
Australasia	3 (9)	1 (3)	2 (23)	1 (20)
Europe	8 (24)	10 (29)	3 (33)	1 (20)
North America	11 (32)	15 (44)	1 (11)	3 (60)
South America	2 (6)	0 (0)	0 (0)	0 (0)

EPDS, Edinburgh Postnatal Depression Scale; BDI, Beck Depression Inventory; CES‐D, Center for Epidemiological Studies‐Depression; PHQ‐9, Patient Health Questionnaire‐9; DASS, Depression Anxiety Stress Scales; MHI‐5, Mental Health Inventory‐5; MADRS, Montgomery–Åsberg Depression Rating Scale; Kessler 6, Kessler 6 Mental Health Scale; Zung SDS, Zung Self‐Rating Depression Scale; STAI, State–Trait Anxiety Inventory.

Over half of the studies (*N* = 38) measured depression with screening tools, with some studies measuring depression at more than one time. The most frequently used tool was the Edinburgh Postnatal Depression Scale (EPDS; *N* = 16), with different cut‐off scores to indicate ‘caseness’ for depression, reflecting different populations. Five of the included studies also used the State–Trait Anxiety Inventory (STAI) as a measure of anxiety. When diagnostic codes were used, these were usually International Classification of Diseases, ninth or tenth revision (ICD‐9 or ICD‐10).

Many of the studies did not provide clear criteria for GDM diagnosis. Ten did not provide any information, eight were self‐report and 16, although appearing to use clinical diagnoses, did not specify diagnostic criteria. Twenty‐eight studies did provide this information. Eight used Carpenter–Coustan criteria for 100 g 3‐h oral glucose tolerance test (OGTT). For studies using 2‐h 75 g OGTT, seven of the studies used current International Association of Diabetes and Pregnancy Study Groups (IADPSG) criteria, two used Australasian Diabetes in Pregnancy Society (ADIPS) criteria and one used 2008 Canadian Diabetes Association criteria. One study used Dutch midwifery and obstetric guidelines and another used Finnish clinical guidelines. Five studies provided specific diagnostic criteria: the origins of which were unclear and three merely reported ‘OGTT’.

Twenty‐four studies were from North America, 16 from Europe, 15 from Asia, four from Australasia, two from South America and one from Africa. Thirteen were upper‐ or lower–middle income countries; none were low‐income countries (according to World Bank classification at June 2018). There were no studies from the UK. The most common study design was a prospective cohort (28 studies). Eighteen studies were cross‐sectional in design, eight were retrospective cohorts, five were intervention studies and three were case–control studies. Thirty‐six studies were assessed as high risk of bias; this was predominantly due to lack of information about how GDM or mental disorder was diagnosed, increasing the risk of measurement bias and/or lack of information about participation rates, exclusion or inclusion criteria preventing accurate assessment of risk of selection bias.

### Odds and prevalence of high levels of antenatal depressive symptoms in women with GDM around the time of GDM diagnosis

Twenty‐seven studies measured levels of depressive symptoms occurring at the time of GDM diagnosis or which were unclear about when in pregnancy diagnosis of mental disorder occurred.

**Figure 3:**
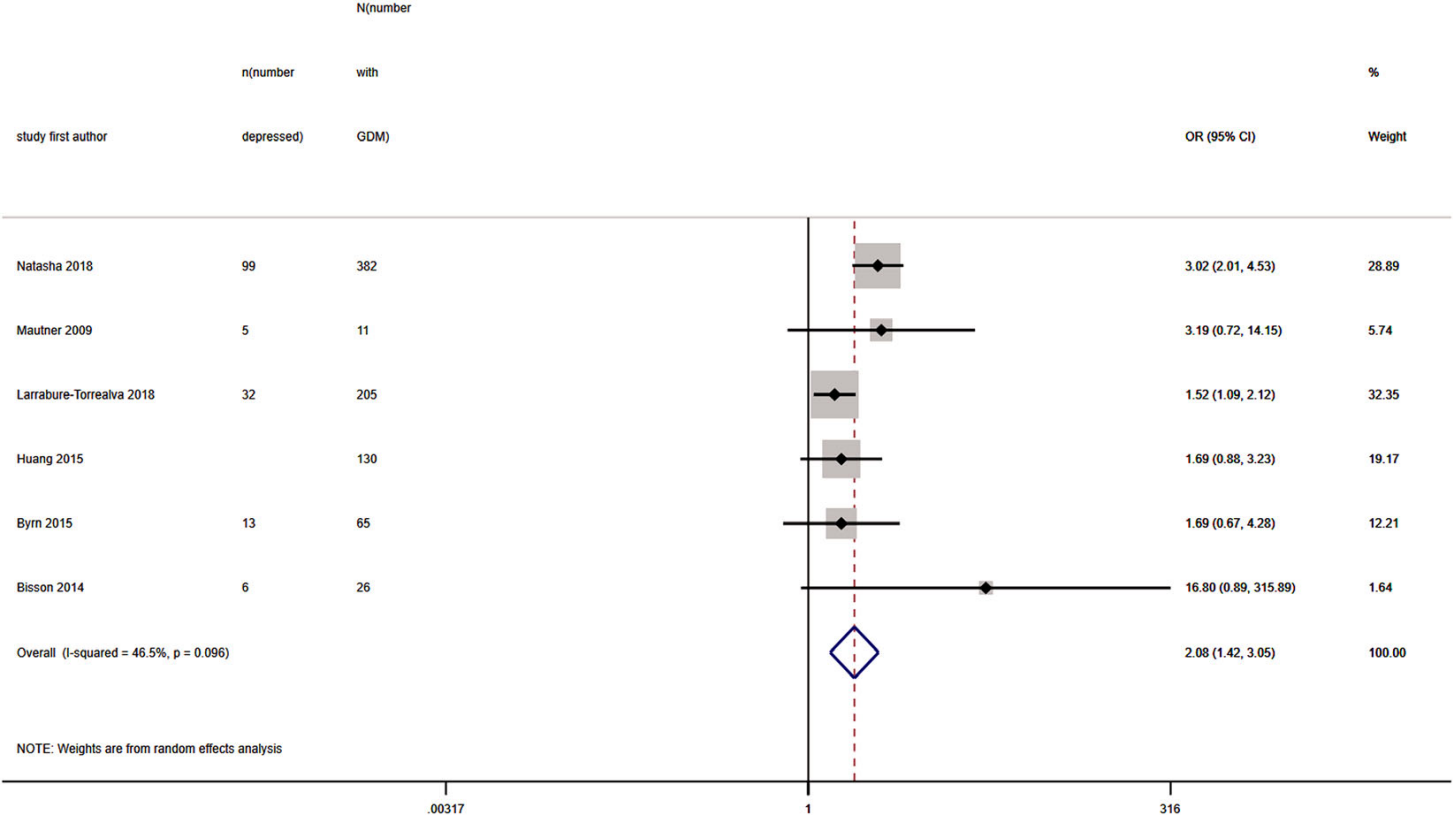
Forest plot showing pooled odds ratios for high levels of antenatal depressive symptoms at the time of GDM diagnosis in women with GDM vs. those without GDM.

Ten studies provided prevalence data at the time of GDM diagnosis (Table 2) but heterogeneity was 97%, precluding meta‐analysis. Median prevalence of high levels of antenatal depressive symptoms in women with GDM was 28% (IQR 20%–46%) (10 studies; *N* = 5515). Six of these studies (*N* = 4387) were also used in a meta‐analysis of unadjusted OR (although age‐adjusted OR was used for one of the studies as unadjusted was not provided), yielding a pooled OR of 2.08 (95% CI 1.42, 3.05) with heterogeneity at 47% (Fig. 3).

**Table 2:** Summary of data provided by each study and effect estimates for high levels of antenatal depressive symptoms at the time of GDM diagnosis

Author and year	Sample size	Mental disorder measure and time	GDM measure	Ethnicity	Pre‐pregnancy BMI (kg/m^2)^	Risk of bias	Prevalence of depression in GDM group (%)	Unadjusted OR (95% CI)
Bisson *et al*., 2014	52	EPDS ≥10 Time of enrolment, mean 30 weeks gestation	75 g OGTT 24–28 weeks' gestation 2008 Canadian Diabetes Association criteria On 75 g OGTT, two or more of: Fasting ≥ 5.3 mmol/l 1‐h ≥ 10.6 mmol/l 2‐h ≥ 8.9 mmol/l	Canadian (ethnicity unspecified)	Mean (sd) GDM: 26.3 (4.5) No GDM: 26.4 (4.5)	High	23.1†	16.8* (0.89–315.89)
Byrn and Penckofer, 2015	135	EPDS ≥ 12 24–40 weeks gestation	‘Medical data to verify GDM status’	White: 32.6% Black: 23% Hispanic: 32.6% Other: 11.9%	Mean (sd) 32.6 (6.42) NB not clear when measured	Low to moderate	20†	1.69* (0.67–4.28)
Dame *et al*., 2017	820	EPDS ≥ 12 Third trimester	Self‐report and confirmed by medical records. Criteria based on two‐step approach. ‘Initially, diagnoses were often made using a single elevated 2‐h plasma glucose test. However, more recently, the criteria developed by the IADPSG have become more commonly used.'	Brazil (ethnicity unspecified)	< 25: 22.8% Overweight: 30.4% Obese class one: 27.6% Obese class two or three: 19.3%	High	31†	Unavailable
Daniells *et al*., 2003	100	MHI‐5 ≥ 16 30 weeks gestation	ADIPS criteria One or more of: Fasting plasma glucose ≥ 5.1 mmol/l 1‐h post 75 g OGTT ≥ 10.0 mmol/l 2‐h post 75 g OGTT ≥ 8.5 mmol/l	Australian born GDM: 66% No GDM: 86%	Mean (sd) GDM: 27.4 (7.2) No GDM: 24.6 (3.8)	High	30†	Not used for meta‐analysis as unclear if pre‐GDM excluded
Ghaffar *et al*., 2016	108	EPDS ≥ 11 13–40 weeks' gestation	OGTT >7 mmol/l	Pakistan (ethnicity unspecified)	Unavailable	High	56.5†	Unavailable
Hassan *et al*., 2017	100	BDI ≥ 20 (unknown version) 24–36 weeks' gestation	75 g OGTT using ADA criteria	Iraq (ethnicity unspecified)	Mean (sd) GDM: 27.4 (7.2) No GDM: 24.6 (3.8) NB not clear when measured	High	86†	Not used for meta‐analysis as unclear if pre‐GDM excluded
Huang *et al*., 2015	2112	EPDS ≥ 13 Time of diagnosis	1‐h 50 g non‐fasting glucose challenge test (GCT) and if > 140 mg/dl (7.8 mmol/l), then 3‐h fasting 100 g OGTT according to ADA criteria	(Percentages across categories of depression) White: 50–72% Black: 13–24% Hispanic: 6–14% Asian: 4–7% Other: 4–7%	(Percentages across categories of depression) Underweight (< 18.5): 3–6% Normal (18.5 to < 25): 50–61% Overweight(25 to < 30): 20–26% Obese (≥ 30): 14–25%	High	12.7†	1.69† (0.88–3.23) (age adjusted)
Larrabure‐Torrealva *et al*., 2018	1300	PHQ‐9 ≥ 10 24–28 weeks gestation	IADPSG criteria Any one of: Fasting plasma glucose ≥ 5.1 1‐h post 75 g OGTT ≥ 10.0 mmol/l 2‐h post 75 g OGTT ≥ 8.5 mmol/l	Mestizo 98.1% Other 1.9%	< 25: 53.6% 25–29.99: 34.9% ≥ 30: 11.5%	High	15.6†	1.52† (1.09–2.12)
Mautner *et al*., 2009	40	EPDS ≥ 10 24–37 weeks gestation	Medical records	Austria (ethnicity unspecified)	Unavailable	High	45.5‡	3.19‡ (0.72–14.15)
Natasha *et al*., 2018	748	MADRS ≥13 24–28 weeks' gestation	Plasma glucose ≥7.0 (WHO criteria) or ≥ 5.3 mmol/l at fasting and ≥ 8.6 mmol/l at 2‐h post 75g OGTT (ACOG criteria)	Bangladesh (ethnicity unspecified)	Unavailable	Low to moderate	25.9†	3.02† (2.01–4.53)
Median prevalence 28% (IQR 20–45.5%)								
Pooled OR 2.08 (95% CI 1.42–3.05)								

*Derived from data in paper. ^†^Estimate given in paper. ^‡^Data provided by study author.

ADIPS, Australasian Diabetes in Pregnancy Society; BDI, Beck Depression Inventory; EPDS, Edinburgh Postnatal Depression Scale; IADPSG, International Association of Diabetes and Pregnancy Study Groups; MADRS, Montgomery–Åsberg Depression Rating Scale; MHI‐5, Mental Health Inventory‐5; OGTT, oral glucose tolerance test; PHQ‐9, Patient Health Questionnaire‐9.

Of the 17 studies not included in meta‐analysis (Tables S2 and S3), there were 11 for which the time of depression measurement in the antepartum in relation to GDM diagnosis could not be ascertained (references 18, 20, 23, 27, 30, 35, 40, 46, 49, 51 and 60 in Appendix S2). One study provided only results stratified by BMI (reference 59 in Appendix S2) and another used depression as exposure not outcome (reference 53 in Appendix S2). Finally, four studies presented only mean scores on depression screening tools; they did not provide data on numbers scoring above and below a specified cut‐off for ‘caseness’ on these tools, preventing calculation of prevalence or odds (references 15, 22, 37 and 39 in Appendix S2).

### Odds and prevalence of high levels of antenatal depressive symptoms in women with GDM following GDM diagnosis

Eight studies (*N* = 862) measured levels of depressive symptoms occurring in the late antepartum following GDM diagnosis (Table 3). Pooled prevalence for high levels of depressive symptoms across all studies was 26% (95% CI 18%, 35%) with heterogeneity at 66%.

**Table 3:** Summary of data provided by each study and effect estimates for high levels of antenatal depressive symptoms following GDM diagnosis

Author and year	Sample size	Mental disorder measure and time	GDM measure	Ethnicity	Pre‐pregnancy BMI (kg/m^2)^	Risk of bias	Prevalence of depression in GDM group (%)	Unadjusted OR (95% CI)
Besser *et al*., 2007	209	CES‐D ≥ 16 After GDM diagnosis	50 g OGTT 24–28 weeks' gestation Two abnormal OGTTs (at 1 and 3 h)	Israel (ethnicity unspecified)	Unavailable	Low to moderate	29†	0.86* (0.48–1.56)
Bublitz *et al*., 2017	24	PHQ‐9 ≥ 10 After GDM diagnosis	1‐ or 3‐h glucose tolerance test	Caucasian: 54%	Mean (sd) 32.54 (7.5) NB: at time of GDM diagnosis	High	12.5†	Unavailable
Chazotte *et al*., 1995	60	CES‐D ≥ 16 34–36 weeks gestation	Not specified	Black: 45% Hispanic: 48.3% White: 6.7%	Unavailable	High	56.7†	2.62* (0.92–7.46)
Keskin *et al*., 2015	89	BDI (unknown version) ≥ 17 After GDM diagnosis	2‐h 75 g OGTT: plasma glucose during fasting ≥ 92 mg/dl (5.1 mmol/l) or at 1 h ≥ 180 mg/dl (10.0 mmol/l) or at 2 h ≥ 153 mg/dl (8.5 mmol/l)	Turkey (ethnicity unspecified)	Unclear when measured GDM: 27.6 (5.8) No GDM: 25.1 (4.3)	High	20.5†	1.19 (0.41–3.43)
Ragland *et al*., 2010	50	BDI‐II ≥ 14 After GDM diagnosis	Not specified	Black: 38% Hispanic: 10% White: 50% Other: 2%	Unavailable	High	40.9†	Unavailable
Rumbold and Crowther, 2002	209	EPDS ≥ 12 After GDM diagnosis	75 g OGTT WHO criteria	Caucasian: 90% Asian: 5% Aboriginal: 1% Other: 5%	Mean (sd) 28 (6)	High	19†	1.03* (0.32–3.36)
Song *et al*., 2004	104	Zung SDS ≥ 41 After GDM diagnosis	75 g OGTT Fasting blood glucose ≥ 6.1 mmol/l or 2‐h plasma glucose ≥ 7.8 mmol/l	China (ethnicity unspecified)	Unavailable	High	22†	3.53* (1.04–11.93)
Varela *et al*., 2017	117	EPDS ≥ 13 32–35 weeks' gestation	Self‐report (not reported in paper but information provided by study author) Diagnostic criteria unknown	Greek 93.2%	Unavailable	High	17.6†	1.73† (0.43‐7)
Pooled prevalence 26% (95% CI 18–35)								
Pooled OR 1.41 (95% CI 0.88–2.25)								

*Derived from data in paper. ^†^Estimate given in paper.

BDI, Beck Depression Inventory; CES‐D, Center for Epidemiological Studies‐Depression; EPDS, Edinburgh Postnatal Depression Scale; OGTT, oral glucose tolerance test; PHQ‐9, Patient Health Questionnaire‐9; WHO, World Health Organization; Zung SDS, Zung Self‐Rating Depression Scale.

**Figure 4:**
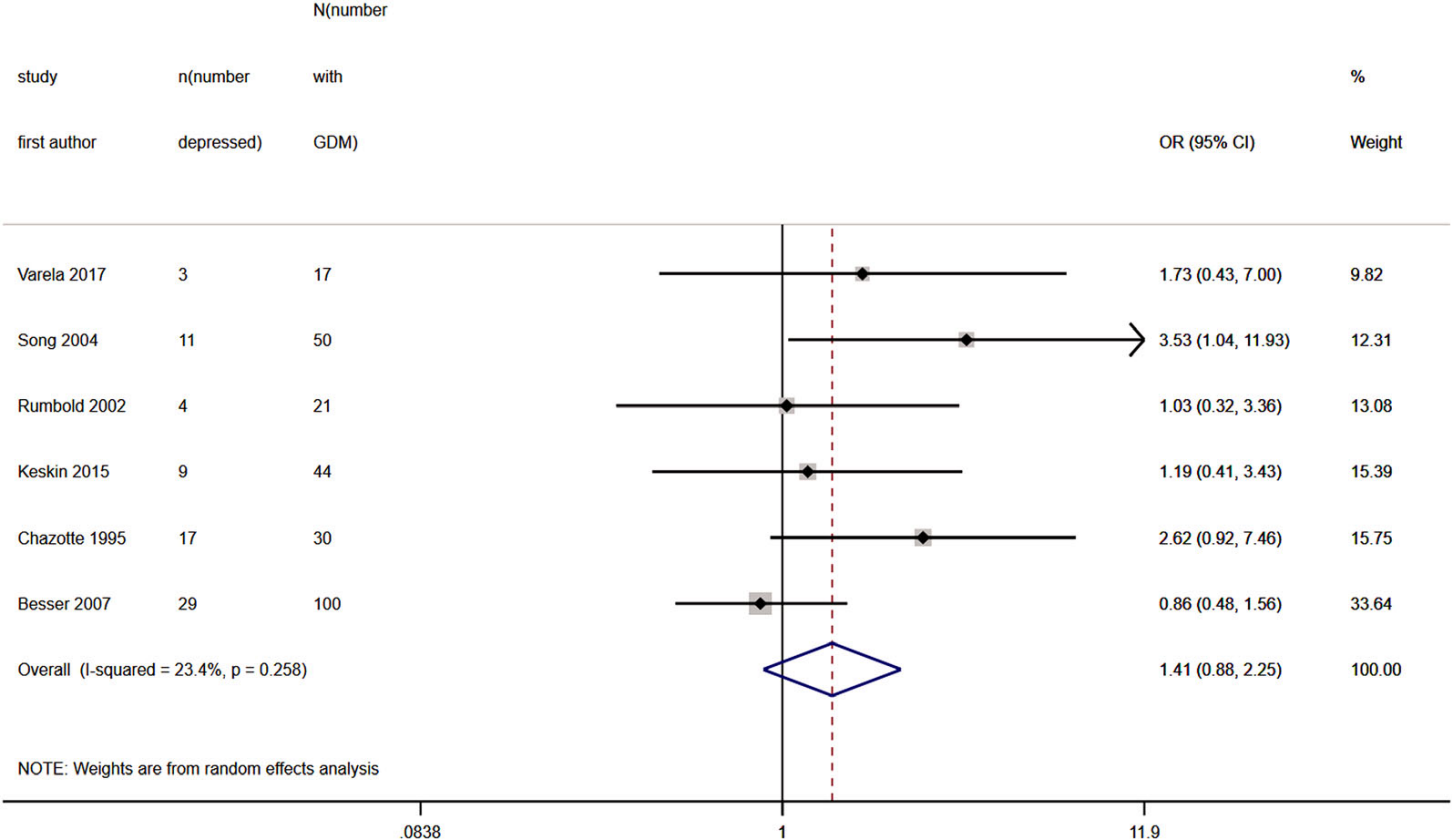
Forest plot showing pooled odds ratios for high levels of antenatal depressive symptoms in women after a diagnosis of GDM vs. those without a diagnosis of GDM.

Six studies (*N* = 788) provided data on unadjusted ORs for high levels of depressive symptoms in those who received and did not receive the GDM diagnosis. Pooled unadjusted OR was 1.41 (95% CI 0.88, 2.25), with heterogeneity at 23% (Fig. 4).

### Odds and prevalence of high levels of postnatal depressive symptoms in women with GDM

Thirty‐four studies measured depression as a diagnosis or levels of depressive symptoms occurring in the postpartum. Twenty‐five studies (*N* = 2 324 634) provided prevalence data (Table 4). Heterogeneity on meta‐analysis was 99% so median prevalence of high levels of postnatal depressive symptoms in women with GDM is presented, which was 13% (IQR 10%–26%).

**Table 4:** Summary of data provided by each study and effect estimates for high levels of postnatal depressive symptoms

Author and year	Sample size	Mental disorder measure and time	GDM measure	Ethnicity	Pre‐pregnancy BMI (kg/m^2)^	Risk of bias	Prevalence of depression in GDM group (%)	Unadjusted OR (95% CI)
Al‐Shahrani *et al*., 2011	113	EPDS ≥12 1 week postpartum	75 g OGTT	Saudi Arabia (ethnicity unspecified)	Unavailable	High	41.1†	Not used for meta‐analysis as unclear if pre‐GDM excluded
Beka *et al*., 2018	326 723	ICD‐9 and ‐10 Up to 1 year postpartum	Diagnostic codes 24–28 weeks gestation	Aboriginal: 5.9% Chinese: 3% South Asian: 8.5% ‘General population’: 82.6%	Underweight (≤ 45kg) 0.6% Overweight (≥ 91kg) 8.6%	Low to moderate	13.1†	1.06* (1.00–1.11)
Bener *et al*., 2012	1379	EPDS ≥12 Within 6 months of delivery	Not specified	Qatar (ethnicity unspecified)	Unavailable	High	25.5†	Not used for meta‐analysis as unclear if pre‐GDM excluded
Berger *et al*., 2015	537	EPDS ≥13 Between day 0 and day 4 postpartum	Not specified	Caucasian: 63.6–71.1% African American: 5.5–9.1% Other: 22.5–29.7%	Mean (sd) From 25.7 (6.0) to 27.6 (8.6) in different subgroups	High	9.7*	Not used for meta‐analysis as unclear if pre‐GDM excluded
Besser *et al*., 2007	209	CES‐D ≥ 16 8 weeks' postpartum	50 g OGTT 24–28 weeks' gestation Two abnormal OGTT (at 1 and 3 h)	Israel (ethnicity unspecified)	Unavailable	Low to moderate	47†	1.31* (0.76–2.27)
Blom *et al*., 2010	4941	EPDS ≥ 13 Two months postpartum	Midwife and hospital registries Dutch midwifery and obstetric guidelines: random glucose > 11.1 mmol/l or fasting > 7.0 mmol/l	Dutch: 62.9% Other Western: 18.7% Non‐Western: 18.4%	Unavailable	Low to moderate	12.5†	Not used for meta‐analysis as unclear if pre‐GDM excluded
Clark *et al*., 2019	766	DSM‐IV major depressive disorder, atypical depressive disorder or depressive disorder not otherwise specified Up to 6 months postpartum	1‐h 75 g OGTT: 7.8 mmol/l 1‐h 100 g OGTT: 10 mmol/l 2‐h 100 g OGTT: 8.45 mmol/l Fasting glucose: 5.1 mmol/l	Caucasian: 40.2% Hispanic: 15.7% African American: 4.5% Asian: 36.8% Native American: 2.8%	Unavailable	Low to moderate	7.9*	0.94 (0.56–1.58)*
Farr *et al*., 2014	4451	Self‐report but validated against diagnostic interview ‘in the postpartum'	Self‐report	USA (unable to calculate)	Unable to calculate	Low to moderate	10.9*	Only adjusted estimates provided
Ferrari *et al*., 2018	173	BDI‐I ≥10 and BDI‐II ≥14 Up to 1 year postpartum	OGTT and IADPSG criteria Any one of: Fasting plasma glucose ≥5.1 mmol/l 1‐h post 75 g OGTT ≥10.0 mmol/l 2‐h post 75 g OGTT ≥8.5 mmol/l	Germany (ethnicity unspecified)	NB in postpartum Mean (sd) No depressive symptoms: 25.5 (5.9) Mild/moderate depressive symptoms 29.2 (7.7)	High	12.7†	Unavailable
Gunderson *et al*., 2015	1035	CES‐D ≥16 6–9 weeks postpartum	Carpenter–Coustan criteria On 3‐h 100g OGTT, two or more of: Fasting ≥ 5.3 mmol/l 1 h ≥ 10 mmol/l 2 h ≥ 8.6 mmol/l 3 h ≥ 7.8 mmol/l	Non‐Hispanic White: 15–24.6% Non‐Hispanic Black: 7–11.5% Hispanic: 29–40.7% Asian: 31.9–37.4% Other: 0.9–2%	Mean (sd) 29 (6.9) to 33.4 (8.3	Low to moderate	13.2†	Unavailable
Hinkle *et al*., 2016	2802	EPDS ≥10 at 6 weeks postpartum or self‐reported antidepressant medication use in postpartum	Medical records Carpenter–Coustan criteria On 3‐h 100 g OGTT, two or more of: Fasting ≥5.3 mmol/l 1‐h ≥ 10 mmol/l 2‐h ≥ 8.6 mmol/l 3‐h ≥ 7.8 mmol/l	Non‐Hispanic White: 28% Non‐Hispanic Black: 27.6% Hispanic: 28.8% Asian: 15.6%	Normal weight: 56.4% Overweight: 26.3% Obese 17.3%	High	14.8†	4.52† (1.23–16.69)
Huang *et al*., 2015	2112	EPDS ≥13 6 months postpartum	1‐h 50 g non‐fasting glucose challenge test (GCT) and if > 140 mg/dl, then 3‐h 100 g fasting OGTT according to ADA criteria	(Percentages across categories of depression) White: 50–72% Black: 13–24% Hispanic: 6–14% Asian: 4–7% Other: 4–7%	(Percentages across categories of depression) Underweight (< 18.5): 3–6% Normal (18.5 to < 25): 50–61% Overweight (25 to < 30): 20–26% Obese (≥ 30): 14–25%	High	11†	1.45† (0.71–2.99) (age adjusted)
Katon *et al*., 2014	1319	PHQ‐9 ≥ 10 6 weeks postpartum	ICD‐9 GDM (648.8) from medical records	White: 73.8% African American: 5.6% Hispanic: 4.6% Other: 16.0%	Unavailable	Low to moderate	5.4†	1.02* (0.57‐1.8)
Kim *et al*., 2005	1445	Short form CES‐D 10 items ≥ 11 8–12 weeks postpartum	Medical records	Latina: GDM 50%, control 34.7% White: GDM 25%, control 34.1% African American: GDM 17.2%, control 16.6% Other: GDM 7.8%, control 14.6%	Normal/underweight (< 25): GDM 34.4%, control 62.5% Overweight (25–29.9): GDM 20.3%, control 22.6% Obese (≥30): GDM 45.3%, control 14.9%	Low to moderate	14.1*	Unavailable
Koutra *et al*., 2016	1037	EPDS ≥13 8 weeks postpartum	3‐h 100 g OGTT Carpenter–Coustan criteria Fasting ≥ 5.3 mmol/l 1‐h ≥ 10 mmol/l 2‐h ≥ 8.6 mmol/l 3‐h ≥ 7.8 mmol/l	Greek: 93% Other: 7%	Mean (sd) EPDS < 13 24.16 (4.87) EPDS ≥13 24.18 (4.76)	Low to moderate	18.4†	Not used for meta‐analysis as unclear if pre‐GDM excluded
Kumpulainen *et al*., 2018	3215	CES‐D ≥ 16 2 and/or 28 weeks postpartum	Medical records 75 g OGTT fasting ≥ 5.1, 1 h 10.0 or 2 h 8.5 mmol/l	Finland (ethnicity unspecified)	In early pregnancy‐Underweight (<18.5): 3.3% Normal 18.5–24.99): 63.9% Overweight (25–29.99): 19.6% Obese (≥ 30): 13.3%	Low to moderate	20.9‡	1.13‡ (0.86–1.49)
Mautner *et al*., 2009	40	EPDS ≥ 10 3–4 months postpartum	Medical records	Austria (ethnicity unspecified)	Unavailable	High	27.3‡	1.8‡ (0.35–9.28)
Natasha *et al*., 2018	734	MADRS ≥ 13 Within 1 week postpartum	Plasma glucose ≥ 7.0 (WHO criteria) or ≥ 5.3 mmol/l at fasting and ≥ 8.6 mmol/l at 2‐h post 75 g OGTT (ACOG criteria)	Bangladesh (ethnicity unspecified)	Unavailable	Low to moderate	12.8†	3.39† (1.86–6.17)
Nehbandani *et al*., 2016	262	EPDS ≥12 4–6 weeks postpartum	At least one abnormal result on 2‐h 75 g OGTT: fasting ≥ 92 mg/dl (5.1 mmol/l) 1‐h ≥ 180 mg/dl (10 mmol/l) 2‐h ≥ 153 mg/dl (8.5 mmol/l)	Iran (ethnicity unspecified)	Unavailable	High	34.3†	2.21* (1.25–3.89)
Nicklas *et al*., 2013	71	EPDS ≥9 4–15 weeks postpartum	Carpenter–Coustan criteria On 3‐h 100g OGTT, two or more of: Fasting ≥ 5.3 mmol/l 1 h ≥ 10 mmol/l 2 h ≥8 .6 mmol/l 3 h ≥ 7.8 mmol/l	African American: 28% Asian: 13% Hispanic: 21% Non‐Hispanic White: 38%	Mean 30	High	33.8†	Unavailable
O'Reilly *et al*., 2016	573	PHQ‐9 ≥ 10 3 months postpartum	ADIPS criteria One or more: Fasting plasma glucose ≥5.1 mmol/l 1‐h post 75 g OGTT ≥ 10.0 mmol/l 2‐h post 75 g OGTT ≥ 8.5 mmol/l	Africa: 3.5% Americas: 0.7% Asia: 38.9% Europe: 29% Oceania: 1.2% Australia and New Zealand: 22.9% Aboriginal and Torres Strait Islander: 0.2%	Mean (sd) 28.8 (6.8) Postnatal baseline	Low to moderate	10.4†	Unavailable
Ruohomäki *et al*., 2018	1066	EPDS ≥ 10 8 weeks postpartum	Medical records Finnish current care guidelines: 75 g OGTT with at least one abnormal value ≥ 5.3 mmol/l for fasting stage, ≥10.0 mmol/l at 1 h and ≥ 8.6 mmol/l at 2 h=	Finnish (ethnicity unspecified)	Mean (sd) No GDM: 24.1 (4.3) GDM: 28.7 (6.5)	High	Unavailable	1.84† (1.13–3.00)
Silverman *et al*., 2017	701 404	ICD‐9 and ‐10 Up to 1 year postpartum	Birth registers	Sweden (ethnicity unspecified)	Unavailable	High	1.4†	2.3* (1.72–3.09)
Varela *et al*., 2017	93	EPDS ≥ 13 1 week postpartum	Self‐report (not reported in paper but information provided by study author)	Greek: 97.8%	Unavailable	High	35.7†	4.32† (1.18–15.78)
Walmer *et al*., 2015	18 888	ICD‐9 ‘in the postpartum’	1‐h 50 g glucose load test ≥ 7.8 and 3‐h 100 g OGTT Carpenter–Coustan criteria On 3‐h 100g OGTT, two or more of: Fasting ≥ 5.3 mmol/l 1 h ≥ 10 mmol/l 2 h ≥ 8.6 mmol/l 3 h ≥ 7.8 mmol/l	Asian: 7.2% Black: 5.8% Hispanic: 25.7% White: 61.3%	Mean (sd) From first antenatal visit GDM: 29 (7.2) No GDM: 25.3 (5.2)	Low to moderate	Unavailable	1.45† (1.15–1.82) (age adjusted)
Youn *et al*., 2017	1 269 130	ICD‐10 Up to 1 year postpartum	ICD‐10 codes	South Korea (ethnicity unspecified)	Unavailable	High	3.9†	Not used for meta‐analysis as unclear if pre‐GDM excluded
Zwolinska‐Kloc *et al*., 2017	70	MINI diagnostic interview ICD‐10 6–7 months postpartum	OGTT 24–28 weeks gestation (not reported in paper but information provided by study author)	Poland (ethnicity unspecified)	Unavailable	High	8.6†	7.65* (0.38 153.76)
Median prevalence 13.1% (IQR 10.4–25.5%)								
Pooled OR 1.59 (95% CI 1.26, 2.00)								

*Derived from data in paper. ^†^Estimate given in paper. ^‡^Data provided by study author.

ADIPS, Australasian Diabetes in Pregnancy Society; BDI, Beck Depression Inventory; CES‐D, Center for Epidemiological Studies‐Depression; DSM‐IV, Diagnostic and Statistical Manual of Mental Disorders‐IV; EPDS, Edinburgh Postnatal Depression Scale; IADPSG, International Association of Diabetes and Pregnancy Study Groups; MADRS, Montgomery–Åsberg Depression Rating Scale; OGTT, oral glucose tolerance test; PHQ‐9, Patient Health Questionnaire‐9.

**Figure 5:**
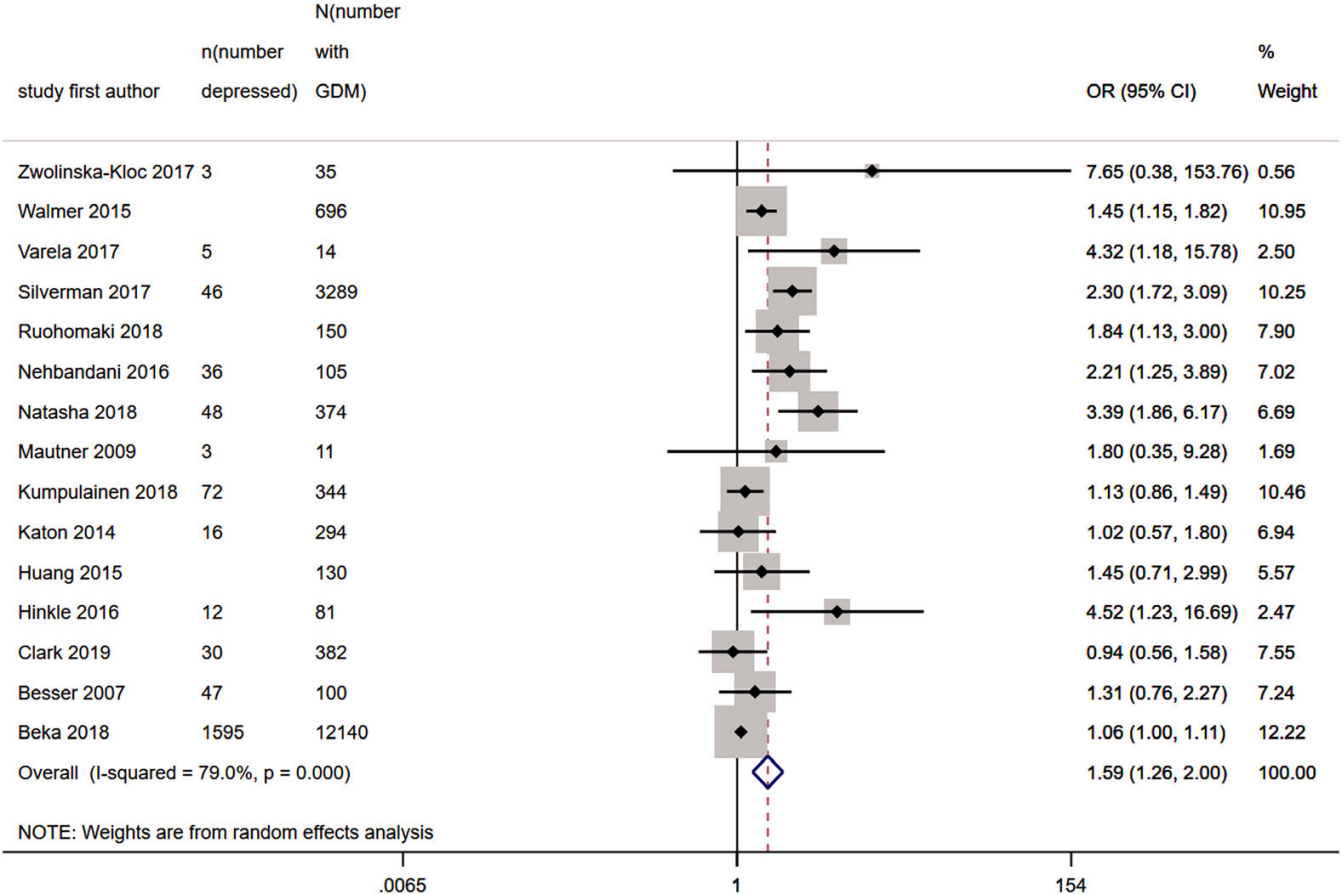
Forest plot showing pooled odds ratios for high levels of postnatal depressive symptoms in women with GDM vs. those without GDM.

Thirteen studies provided unadjusted ORs for high levels of postnatal depressive symptoms in women with GDM vs. those without GDM. Two further studies provided age‐adjusted estimates which were used as an unadjusted estimate was unavailable (Table 4). Pooled OR for these 15 studies (*N* = 1 059 703) was 1.59 (95% CI 1.26, 2.00), with heterogeneity at 79% (Fig. 5).

**Figure 6:**
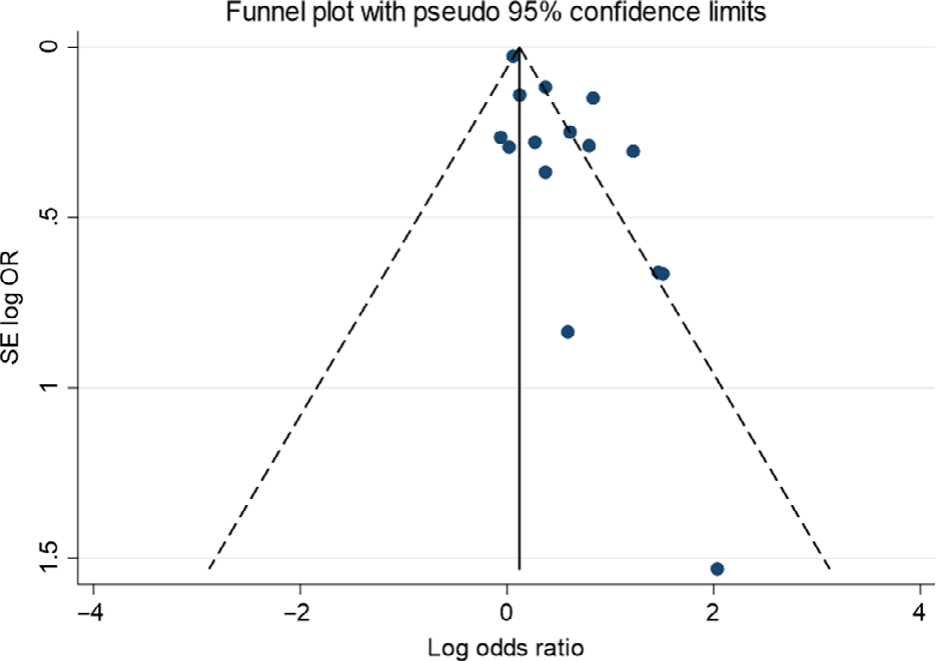
Funnel plot of association between study size and effect size in those studies used in meta‐analysis of odds ratios for postnatal depression.

On visual inspection of the funnel plot including studies in the meta‐analysis of ORs, there was some possible asymmetry, with some missing studies in the bottom left corner (Fig. 6) and Egger's test suggested a significant small study effect (*P* = 0.003).

Seven studies were not included in the meta‐analysis because they presented only incidence data (references 6, 42 and 49 in Appendix S2), continuous EPDS scores (reference 39 in Appendix S2) or ORs that could not be used as it was unclear whether pregestational diabetes had been excluded from the control population and from which prevalence could not be calculated (references 1, 38 and 56 in Appendix S2; see also Tables S2 and S3).

### Sensitivity analyses

Studies at high risk of bias were removed in the postnatal meta‐analysis (Table 5) but there were insufficient studies with low to moderate risk of bias in the antenatal subgroups to facilitate this. This gave median prevalence (12 studies; *N* =1 970 534) of 13% (IQR 11%–16%) and pooled OR (seven studies; *N* = 351 854) of 1.27 (95% CI 1.02, 1.57) with heterogeneity at 72%. Another sensitivity analysis in the postpartum removed six studies (references 3, 14, 54, 58, 61 and 62 in Appendix S2) that used diagnostic as opposed to screening measures. This gave median prevalence (20 studies; *N* = 26 541) of 14% (IQR 12%–31%) and pooled OR (10 studies; *N* = 11 852) of 1.75 (95% CI 1.29, 2.37) with heterogeneity at 57%. In the antenatal meta‐analyses, there were no studies that utilized diagnostic tools at the time of GDM diagnosis or following it. Cumulative meta‐analysis assessing the impact of study sample size on pooled ORs in each of the three meta‐analyses suggested no significant impact of larger studies (Fig. S1).

**Table 5:** Summary of data provided by each study and effect estimates for antenatal and postnatal anxiety symptoms and disorders

Author and year	Sample size	Mental disorder measure and time	GDM measure	Ethnicity	Pre‐pregnancy BMI (kg/m^2^)	Risk of bias	Prevalence of anxiety in GDM group (%)	Unadjusted OR (95% CI)
Antepartum any time during pregnancy or around the time of GDM diagnosis								
Boggaram *et al*., 2017	100	MINI structured interview During pregnancy	‘Diagnosis by trained OB/GYN’ (not reported in paper but information provided by study author)	India (ethnicity unspecified)	Unavailable	High	27.3‡	Unclear if pre‐GDM excluded
Egan *et al*., 2017	218	DASS 21 Anxiety score ≥ 10	Not specified	Caucasian: 89.7–98.1% Non‐Caucasian: 3.1–9%	Unavailable	High	57.7†	1.36* (0.76–2.45)
Hassan *et al*., 2017	100	Taylor anxiety scale ≥25 (moderate to severe anxiety) 24–36 weeks gestation	75 g OGTT using ADA criteria	Iraq (ethnicity unspecified)	Mean (sd) GDM: 27.4 (7.2) No GDM‐: 24.6 (3.8) NB not clear when measured	High	86†	Unclear if pre‐GDM excluded
Postpartum								
Beka *et al*., 2018	326 723	ICD‐9 and ‐10 Up to 1 year postpartum	Diagnostic codes 24‐28 weeks gestation	Aboriginal: 5.9% Chinese: 3% South Asian: 8.5% ‘General population’: 82.6%	Underweight (≤ 45 kg): 0.6% Overweight: (≥ 91 kg) 8.6%	Low to moderate	14.6†	1.08* (1.03–1.14)
Farr *et al*., 2014	4451	Self‐report but validated against diagnostic interview ‘in the postpartum’	Self‐report	USA (unable to calculate)	Unable to calculate	Low to moderate	18.4*	Only adjusted estimates provided
Walmer *et al*., 2015	18 888	ICD‐9 ‘in the postpartum’	1‐h 50 g glucose load test ≥ 7.8 and 3‐h 100 g OGTT Carpenter–Coustan criteria On 3‐h 100 g OGTT, two or more of: Fasting ≥ 5.3 mmol/l 1 h ≥ 10 mmol/l 2‐h ≥ 8.6 mmol/l 3‐h ≥ 7.8 mmol/l	Asian: 7.2% Black: 5.8% Hispanic: 25.7% White: 61.3%	Mean (sd) From first antenatal visit GDM: 29 (7.2) No GDM: 25.3 (5.2)	Low to moderate	Unavailable	1.36† (1.03–1.79) (age adjusted)

*Derived from data in paper. ^†^Estimate given in paper. ^‡^Data provided by study author.

DASS, Depression Anxiety Stress Scales; ICD, International Classification of Disorders; OGTT, oral glucose tolerance test.

### Anxiety symptoms and disorders

Three studies provided data on the prevalence or odds of high levels of anxiety symptoms or anxiety disorders at any time during pregnancy or around the time of GDM diagnosis, and three in the postpartum. These results are summarized in Table 5.

There were a further five studies in which STAI was used and one in which the Depression Anxiety Stress Scales (DASS) was used (reference 22 in Appendix S2); data were presented as continuous scores, precluding calculation of odds or prevalence (Table S3). Four studies utilized the STAI at any time during pregnancy or around the time of GDM diagnosis (references 12, 17, 37 and 43 in Appendix S2). Two of these studies of around 100 women examined differences in STAI state anxiety scores around the time of GDM diagnosis between those with and without GDM; one found significantly higher scores in GDM (reference 17 in Appendix S2) and the other did not (reference 37 in Appendix S2). The study with statistically significant differences followed up women in the late antepartum and postpartum but found no significant differences between women with and without GDM at these points in the peripartum (reference 17 in Appendix S2). Another study post GDM diagnosis also found no significant differences (reference 42 in Appendix S2). However, a Danish population‐based cohort found a statistically significant incidence rate ratio for postpartum reactions to severe stress (ICD‐10) in GDM of 1.42 (95% CI 1.03, 1.97) (reference 52 in Appendix S2).

## Discussion

### Main findings

This is the first study that has meta‐analysed data from studies examining a range of mental disorders throughout the peripartum. We found that the prevalence of high levels of depressive symptoms around the time of GDM diagnosis was 28% (pooled OR 2.08) and following diagnosis was 26% (pooled OR 1.41). This is higher than that expected in the general pregnant population 26, 27. In the postpartum, the prevalence of depression in women diagnosed with GDM during pregnancy was 13% (pooled OR 1.59). This is similar to a recent meta‐analysis which found a pooled relative risk for postnatal depression in women with GDM of 1.59 9. However, the studies included in the two reviews differ slightly, due to stricter inclusion and exclusion criteria in our review, such as ensuring the exclusion of pregestational diabetes from control groups and also a more recent literature search in our study.

It is surprising that there were not more studies that measured levels of anxiety, although there was some evidence for significantly higher anxiety scores in women at the time of GDM diagnosis but no evidence for an increase in anxiety following diagnosis in the antepartum (albeit only two studies identified). There was some evidence for increased odds of postnatal anxiety in women with GDM. Moreover, there is clearly a significant degree of comorbidity between anxiety and depression, and indeed, most of the studies that measured both depression and anxiety generally yielded consistent findings between both disorders, whether it be an increased risk for both in the antepartum (reference 26 in Appendix S2) or postpartum (reference 3 in Appendix S2) or no evidence for an increased risk for either depression or anxiety in the antepartum (reference 18 in AppendixS2) or postpartum (reference 19 in Appendix S2).

### Potential mechanisms

The potential mechanisms underlying the link between GDM and mental disorders are unknown. The type 2 diabetes literature describes potential biological and psychosocial risk factors. Cytokines, part of the inflammatory response, are raised in both depression and type 2 diabetes 28 and can cause pancreatic β‐cell destruction, leading to insulin resistance. There is now growing evidence supporting an inflammatory process in individuals with perinatal depression 29. Cytokines also activate the hypothalamic–pituitary–adrenal axis which regulates the body's response to stress. There may also be an increased risk of hypothyroidism in GDM, which is known to be associated with depression 30.

Psychological factors include the burden of managing a medical condition during pregnancy, which may increase the risk of developing a perinatal mental disorder. Qualitative research exploring women's experiences of GDM has highlighted the strong but widely differing emotional responses to the diagnosis including shock, tearfulness and guilt. Major changes to lifestyle may also be required including dietary changes and blood glucose monitoring 31.

### Strengths and limitations

This review provides a comprehensive synthesis of the literature to date on the association between GDM and mental disorders following diagnosis of GDM. That analyses were grouped by stage of the peripartum is a particular strength of this review because symptoms of mental disorder may fluctuate throughout the peripartum 32. Significant efforts were made to obtain raw data for the meta‐analysis. Assessment of risk of bias allowed the influence of this on the results of meta‐analysis to be considered within sensitivity analysis. Over half of the 62 studies were assessed as at high risk of bias.

Removal of studies at high risk of bias from the postnatal meta‐analyses reduced the effect estimates and there was also some evidence of small study bias. One of the potential sources of bias was measurement bias. All of the mental disorder screening tools used were validated but only indicate ‘caseness’ for mental disorder; they are not diagnostic. There is also little consensus on optimal cut‐off scores for most of the tools. Thus, it is perhaps unsurprising that effect estimates increased when studies using diagnostic codes were removed in sensitivity analyses. However, it is also noteworthy that all three studies contributing to the postnatal meta‐analysis that used diagnostic codes for depression (that also had sample sizes > 10 000) gave ORs that were statistically significant, albeit one was of borderline significance (references 7, 54 and 58 in Appendix S2).

There is also substantial heterogeneity in how GDM is defined (see tables). Unfortunately, many of the studies did not report this. There is some evidence for a relationship between blood glucose below diagnostic threshold for GDM and depression and anxiety 33. Indeed, since the seminal Hyperglycaemia and Adverse Pregnancy Outcomes (HAPO) study observed a linear relationship between elevated maternal glucose concentrations below that of overt diabetes and adverse outcomes, many have argued that GDM should be conceptualized as a continuum of dysglycaemia 34. Recognizing the heterogeneity of GDM, authors of studies in which it seemed likely that results of GDM testing may be available were asked if they would be willing to provide this raw data. However, only two authors felt able to share, precluding any meaningful analysis.

However, management of GDM with insulin may be an indicator of its severity and four of the included studies compared risk for depression between different treatment modalities. Two found no difference in risk for antenatal depression between women managing their GDM with or without insulin (references 15 and 16 in Appendix S2), whereas another study did find an increased risk for postnatal depression in women using insulin (reference 45 in Appendix S2) but another study in the postpartum did not (reference 47 in Appendix S2). Clearly an alternative mechanism for any potential increased risk beyond insulin use being a marker of GDM severity is that its injection merely represents an additional stressor for women.

Further limitations relate to unmeasured confounding in the included studies. As most of the studies were observational, there are a number of important confounders which could limit causal inference. Most studies provided only unadjusted estimates; estimates adjusted for important confounders were limited. A key confounder when considering the direction of relationships is early pregnancy or pre‐pregnancy mental disorder and use of psychotropic medication; timing of mental disorder measurement may not capture timing of onset. A few studies excluded mental disorder prior to pregnancy and five adjusted for pre‐pregnancy mental disorder, which led to significant attenuation of results in two studies (references 51 and 61 in Appendix S2). Another confounder is obesity 1, 12. The intention was to conduct subgroup analyses by BMI but while a number of studies provided data on BMI distribution (see tables), insufficient data on effect estimates by BMI were available to facilitate this. Adjustment for BMI reduced effect estimates in one study (reference 58 in Appendix S2) but not in others (references 36, 53 and 59 in Appendix S2). There may also be ethnic differences in risk for GDM 35 and mental disorder 36. While a number of included studies reported the ethnicity of their population (see tables), only two studies in the meta‐analyses provided ethnicity adjusted ORs (references 29 and 36 in Appendix S2) and two stratified by ethnicity (references 38 and 58 in Appendix S2). Finally, this review only considers the risk for mental disorders following GDM. Future reviews would usefully examine the risk for GDM in women with mental disorders, particularly given some (albeit limited) emerging evidence for an increased risk of GDM in those using antipsychotics 37, 38.

### Implications

There are a number of examples of effective integration of both physical and mental healthcare, including in the type 2 diabetes population 39. All healthcare professionals in contact with women with GDM, from diabetologists, to obstetricians, to midwives and health visitors, could ask about mental health during contacts with health services in the perinatal period. Current UK National Institute for Health and Care Excellence (NICE) guidelines suggest that professionals consider asking all women two questions about low mood and loss of interest (the Whooley questions) as part of a general discussion about the woman's wellbeing in the antepartum and postpartum 27. Moreover, recent US recommendations on screening for perinatal depression have recommended asking women not only about depressive symptoms, but also about associated risk factors 40. The results of our review suggest that GDM may be considered one of these risk factors, emphasizing the importance of this enquiry at every contact and that women with GDM may require additional support during pregnancy and in the postpartum. This may involve liaison with primary care or psychiatry services.

There may be a perception that women with mental disorder may be less likely to engage with support offered, whether it be for physical or mental health. However, in a recent randomized controlled trial of an intervention in obese pregnant women to reduce gestational weight gain, depression was not associated with poorer adherence 41. Moreover, recognition and treatment of mental disorder in women with GDM may lead to improved outcomes. In type 2 diabetes, there is some evidence that treatment of depression is associated with improved glycaemic control 42 and in GDM, a cross‐sectional relationship has been observed between levels of depressive symptoms and glycaemic control 43 and compliance with GDM therapy 44. Severity of hyperglycaemia during pregnancy may influence future risk of type 2 diabetes in the mother and risk of metabolic syndrome 1 and adverse neurobehavioural outcomes in the child 2. Thus, the potential benefits of providing mental healthcare to women with GDM are numerous and far‐reaching.

In conclusion, this systematic review and meta‐analysis found an increased risk of probable antenatal and postnatal depression (and possibly anxiety) in women with GDM. Future research would usefully focus on risk for other mental disorders, including those occurring prior to pregnancy and in early pregnancy prior to the onset of GDM, and on exploring possible mechanisms.

## Funding sources

CW carried out this work as part of a Medical Research Council (MRC) funded Clinical Research Training Fellowship (MR/P019293/1).

## Competing interests

None.

## Supporting information


**Appendix S1.** Search terms used in Medline, PsycINFO, EMBASE, CINAHL and Cochrane Library.
**Appendix S2.** List of final included studies.
**Table S1.** Risk of bias assessment tool.
**Table S2.** Study characteristics, prevalence and odds ratios grouped by mental disorder and time period.
**Table S3.** Characteristics of studies measuring GDM and mental disorder but data not presented as prevalence or odds ratios.
**Figure S1.** Forest plots showing the impact of sensitivity analyses using a leave one out approach based on sample size.
