# Supplementary material for: Is there an increased risk of perinatal mental disorder in women with gestational diabetes? A systematic review and meta‐analysis
Source: Diabet Med. 2019 Nov 29;37(4):602–22. doi: 10.1111/dme.14170 (PMC7154542; doi:10.1111/dme.14170)
Supplement: Supplementary file 1 — Appendix S1. Search terms used in Medline, PsycINFO, EMBASE, CINAHL and Cochrane Library. Appendix S2. List of final included studies. Table S1. Risk of bias assessment tool. Table S2. Study characteristics, prevalence and odds ratios grouped by mental disorder and time period. Table S3. Characteristics of studies measuring GDM and mental disorder but data not presented as prevalence or odds ratios. Figure S1. Forest plots showing the impact of sensitivity analyses using a leave one out approach based on sample size. [file DME-37-602-s001.docx]

**Appendix S1 Search terms used in Medline, PsycINFO, EMBASE, CINAHL and Cochrane Library**

1. MeSH terms- diabetes mellitus, gestational (in CINAHL), diabetes, gestational (in Cochrane Library and Medline), gestational diabetes (in PsycINFO).
2. gestation* adj2 diabet*
3. GDM
4. 1 OR 2 OR 3
5. Exploded MeSH terms- mental disorders (in CINAHL, Cochrane Library, Medline and PsycINFO) and mental disease (in EMBASE)
6. MeSH terms- mental health (in CINAHL, Cochrane Library, EMBASE, Medline and PsycINFO)
7. psych* adj2 (problem* OR disorder* OR ill* OR health)
8. mental adj2 (problem* OR disorder* OR ill* OR health)
9. anxiety adj2 (problem* OR disorder*)
10. ”stress disorder”
11. phobi*
12. panic
13. obsessi*
14. compulsi*
15. OCD
16. ”obsessive compulsive disorder”
17. PTSD
18. somat* adj disorder
19. “adjustment disorder”
20. dissociat* adj disorder
21. schizo*
22. psychosis
23. psychotic
24. delusion* adj disorder
25. mood adj2 (problem* OR disorder*)
26. affective adj2 (problem* OR disorder*)
27. BPAD
28. bipolar
29. mania
30. manic
31. cyclothymi*
32. depression
33. depressive
34. dysthymi*
35. Exploded MeSH terms- eating disorder(s) (in CINAHL, EMBASE and PsycINFO), feeding and eating disorders (in Cochrane Library and Medline)
36. binge adj eat*
37. anorex*
38. bulimi*
39. eat* adj2 disor*
40. compulsive adj2 (eat* OR vomit* OR purg*)
41. 5 OR 6 OR 7 OR 8 OR 9 OR 10 OR 11 OR 12 OR 13 OR 14 OR 15 OR 16 OR 17 OR 18 OR 19 OR 20 OR 21 OR 22 OR 23 OR 24 OR 25 OR 26 OR 27 OR 28 OR 29 OR 30 OR 31 OR 32 OR 33 OR 34 OR 35 OR 36 OR 37 OR 38 OR 39 OR 40
42. 4 AND 41

**Appendix S2 List of final included studies**

1. Abdollahi F, Zarghami M, Azhar MZ, Sazlina SG, Lye MS. Predictors and incidence of post-partum depression: a longitudinal cohort study. *Journal of Obstetrics & Gynaecology Research* 2014; **40**:2191-2200.

2. Al-Shahrani MS, Al-Sunaidi M, Al-Amri H, Al-Maswary S, Al-Gelban K. Gestational diabetes and postpartum depression. *Arab Journal of Psychiatry* 2011; **22**:133-137.

3. Beka Q, Bowker S, Savu A, Kingston D, Johnson JA, Kaul P. Development of Perinatal Mental Illness in Women With Gestational Diabetes Mellitus: A Population-Based Cohort Study. *Canadian Journal of Diabetes* 2018; **42**:350-355.e351.

4. Bener A, Burgut FT, Ghuloum S, Sheikh J. A Study of Postpartum Depression in a Fast Developing Country: Prevalence and Related Factors. *The International Journal of Psychiatry in Medicine* 2012; **43**:325-337.

5. Berger E, Wu A, Smulian EA, Quiñones JN, Curet S, Marraccini RL*, et al.* Universal versus risk factor-targeted early inpatient postpartum depression screening. *The Journal of Maternal-Fetal & Neonatal Medicine* 2015; **28**:739-744.

6. Bernstein JA, Quinn E, Ameli O, Craig M, Heeren T, McCloskey L*, et al.* Follow-up after gestational diabetes: A fixable gap in women's preventive healthcare. *BMJ Open Diabetes Research and Care* 2017; **5**.

7. Besser A, Priel B, Flett GL, Wiznitzer A. Linear and nonlinear models of vulnerability to depression: Personality and postpartum depression in a high risk population. *Individual Differences Research* 2007; **5**:1-29.

8. Bisson M, Sériès F, Giguère Y, Pamidi S, Kimoff J, Weisnagel SJ*, et al.* Gestational Diabetes Mellitus and Sleep-Disordered Breathing. *Obstetrics & Gynecology* 2014; **123**:634-641.

9. Blom E, Jansen P, FC V, A H, H R, VWV J*, et al.* Perinatal complications increase the risk of postpartum depression. The Generation R Study. *BJOG: An International Journal of Obstetrics & Gynaecology* 2010; **117**:1390-1398.

10. Boggaram SA, Manikanta TS, Maheswari E, Singh H. An exploratory study of identification of psychiatric disorders during pregnancy. *Minerva Psichiatrica* 2017; **58**:203-208.

11. Bublitz M, Martin S, Larson L, Bourjeily G, De La Monte S. Childhood maltreatment and inflammation among pregnant women with gestational diabetes mellitus: A pilot study. *Obstetric Medicine* 2017; **10**:120-124.

12. Byrn M, Penckofer S. The relationship between gestational diabetes and antenatal depression. *Journal of Obstetric, Gynecologic, & Neonatal Nursing* 2015; **44**:246-255.

13. Chazotte C, Freda MC, Elovitz M, Youchah J. Maternal depressive symptoms and maternal-fetal attachment in gestational diabetes. *Journal of Women's Health* 1995; **4**:375-380.

14. Clark CE, Rasgon NL, Reed II DE, Robakis TK. Depression Precedes, But Does Not Follow, Gestational Diabetes. *Acta Psychiatrica Scandinavica* 2019; **139**:311-321.

15. Dalfra MG, Nicolucci A, Bisson T, Bonsembiante B, Lapolla A, Qlisg. Quality of life in pregnancy and post-partum: a study in diabetic patients. *Quality of Life Research* 2012; **21**:291-298.

16. Dame P, Cherubini K, Goveia P, Pena G, Nunes MA, Galliano L*, et al.* Depressive Symptoms in Women with Gestational Diabetes Mellitus: The LINDA-Brazil Study. *Journal of Diabetes Research* 2017; **2017**.

17. Daniells S, Grenyer BFS, Davis WS, Coleman KJ, Burgess JP, Moses RG. Gestational diabetes mellitus: is a diagnosis associated with an increase in maternal anxiety and stress in the short and intermediate term? *Diabetes Care* 2003; **26**:385-389.

18. Egan AM, Dunne FP, Lydon K, Conneely S, Sarma K, McGuire BE. Diabetes in pregnancy: worse medical outcomes in type 1 diabetes but worse psychological outcomes in gestational diabetes. *QJM: An International Journal of Medicine* 2017; **110**:721-727.

19. Farr SL, Dietz PM, O'Hara MW, Burley K, Ko JY. Postpartum Anxiety and Comorbid Depression in a Population-Based Sample of Women. *Journal of Women's Health* 2014; **23**:120-128.

20. Ferrara A, Hedderson MM, Brown SD, Albright CL, Ehrlich SF, Tsai A-L*, et al.* The Comparative Effectiveness of Diabetes Prevention Strategies to Reduce Postpartum Weight Retention in Women With Gestational Diabetes Mellitus: The Gestational Diabetes’ Effects on Moms (GEM) Cluster Randomized Controlled Trial. *Diabetes Care* 2016; **39**:65-74.

21. Ferrari U, Banning F, Freibothe I, Trondle K, Sacco V, Wichmann C*, et al.* Depressive symptoms, impaired glucose metabolism, high visceral fat, and high systolic blood pressure in a subgroup of women with recent gestational diabetes. *Journal of Psychiatric Research* 2018; **97**:89-93.

22. Fiskin G, Sahin NH. Effect of diaphragmatic breathing exercise on psychological parameters in gestational diabetes: A randomised controlled trial. *European Journal of Integrative Medicine* 2018; **23**:50-56.

23. Gemeay EM, Moawed SA, Mansour EA, Ebrahiem NE, Moussa IM, Nadrah WO. The association between diabetes and depression. *Saudi Medical Journal* 2015; **36**:1210-1215.

24. Ghaffar S, Rizvi S, Saeed G, Jafri SMH, Jafri A, Haider R. Frequency of depression among hospitalized pregnant females for obstetrics risk. *Pakistan Journal of Medical and Health Sciences* 2016; **10**:898-902.

25. Gunderson EP, Hurston SR, Ning X, et al. Lactation and progression to type 2 diabetes mellitus after gestational diabetes mellitus: A prospective cohort study. *Annals of Internal Medicine* 2015; **163**:889-898.

26. Hassan SM, Harba U, Ejerish MA. Effect of depression and anxiety on gestational diabetes in Babylon government. *International Journal of Pharmaceutical Sciences and Research* 2017; **8**:4371-4375.

27. Hermon N, Wainstock T, Sheiner E, Golan A, Walfisch A. Impact of maternal depression on perinatal outcomes in hospitalized women—a prospective study. *Archives of Women's Mental Health* 2018.

28. Hinkle SN, Buck Louis GM, Rawal S, Zhu Y, Albert PS, Zhang C. A longitudinal study of depression and gestational diabetes in pregnancy and the postpartum period. *Diabetologia* 2016; **59**:2594-2602.

29. Huang T, Rich-Edwards J, James-Todd T, Gillman MW, Oken E, Rifas-Shiman SL*, et al.* Pregnancy hyperglycaemia and risk of prenatal and postpartum depressive symptoms. *Paediatric and Perinatal Epidemiology* 2015; **29**:281-289.

30. Katon JG, Russo J, Gavin AR, Melville JL, Katon WJ. Diabetes and depression in pregnancy: is there an association? *Journal of Women's Health* 2011; **20**:983-989.

31. Katon W, Russo J, Gavin A. Predictors of Postpartum Depression. *Journal of Women's Health (15409996)* 2014; **23**:753-759.

32. Keskin FE, Ozyazar M, Pala AS, Elmali AD, Yilmaz B, Uygunoglu U*, et al.* Evaluation of cognitive functions in gestational diabetes mellitus. *Experimental & Clinical Endocrinology & Diabetes* 2015; **123**:246-251.

33. Kim C, Brawarsky P, Jackson RA, Fuentes-Afflick E, Haas JS. Changes in health status experienced by women with gestational diabetes and pregnancy-induced hypertensive disorders. *Journal of Women's Health* 2005; **14**:729-736.

34. Koutra K, Vassilaki M, Georgiou V, Koutis A, Bitsios P, Kogevinas M*, et al.* Pregnancy, perinatal and postpartum complications as determinants of postpartum depression: the Rhea mother–child cohort in Crete, Greece. *Epidemiology and Psychiatric Sciences* 2016; **27**:244-255.

35. Kumpulainen SM, Girchenko P, Lahti-Pulkkinen M, Reynolds RM, Tuovinen S, Pesonen A-K*, et al.* Maternal early pregnancy obesity and depressive symptoms during and after pregnancy. *Psychological Medicine* 2018; **48**:2353-2363.

36. Larrabure-Torrealva GT, Martinez S, Luque-Fernandez MA, Sanchez SE, Mascaro PA, Ingar H*, et al.* Prevalence and risk factors of gestational diabetes mellitus: findings from a universal screening feasibility program in Lima, Peru. *BMC Pregnancy and Childbirth* 2018; **18**:303.

37. Levy-Shiff R, Lerman M, Har-Even D, Hod M. Maternal adjustment and infant outcome in medically defined high-risk pregnancy. *Developmental Psychology* 2002; **38**:93.

38. Liu CH, Tronick E. Rates and predictors of postpartum depression by race and ethnicity: results from the 2004 to 2007 New York City PRAMS survey (Pregnancy Risk Assessment Monitoring System). *Maternal & Child Health Journal* 2013; **17**:1599-1610.

39. Mak JKL, Lee AH, Pham NM, Tang L, Pan X-F, Binns CW*, et al.* Gestational diabetes and postnatal depressive symptoms: A prospective cohort study in Western China. *Women and Birth* 2018.

40. Manoudi F, Chagh R, Benhima I, Asri F, Diouri A, Tazi I. [Depressive disorders in diabetic patients]. *Encephale* 2012; **38**:404-410.

41. Mautner E, Greimel E, Trutnovsky G, Daghofer F, Egger JW, Lang U. Quality of life outcomes in pregnancy and postpartum complicated by hypertensive disorders, gestational diabetes, and preterm birth. *Journal of Psychosomatic Obstetrics & Gynecology* 2009; **30**:231-237.

42. Meltzer-Brody S, Maegbaek ML, Medland SE, Miller WC, Sullivan P, Munk-Olsen T. Obstetrical, pregnancy and socio-economic predictors for new-onset severe postpartum psychiatric disorders in primiparous women. *Psychological Medicine* 2017; **47**:1427-1441.

43. Miazgowski T, Bikowska M, Ogonowski J, Taszarek A. The Impact of Health Locus of Control and Anxiety on Self-Monitored Blood Glucose Concentration in Women with Gestational Diabetes Mellitus. *Journal of Women's Health* 2018; **27**:209-215.

44. Natasha K, Azad Khan A. Depression, Gestational Diabetes Mellitus and the Impact on Pregnancy Outcomes: A Hospital based study from Bangladesh. . *American J Epidemiol Public Health* 2018; **2**:001-009.

45. Nehbandani S, Nahidi F, Kariman N, Nasiri M. Relationship between gestational diabetes and postpartum depression. *Iranian Journal of Obstetrics, Gynecology and Infertility* 2016; **19**:18-24.

46. Ng SK, Scuffham PA, Cameron CM, Hills AP, McClure RJ. Socioeconomic disparities in prepregnancy BMI and impact on maternal and neonatal outcomes and postpartum weight retention: The EFHL longitudinal birth cohort study. *BMC Pregnancy and Childbirth* 2014; **14**.

47. Nicklas J, Miller L, Zera C, Davis R, Levkoff S, Seely E. Factors Associated with Depressive Symptoms in the Early Postpartum Period Among Women with Recent Gestational Diabetes Mellitus. *Maternal & Child Health Journal* 2013; **17**:1665-1672.

48. O'Reilly SL, Dunbar JA, Carter R, Shih STF, Versace V, Coates M*, et al.* Mothers after Gestational Diabetes in Australia (MAGDA): A Randomised Controlled Trial of a Postnatal Diabetes Prevention Program. *PLoS Medicine* 2016; **13**.

49. Pace R, Rahme E, Da Costa D, Dasgupta K. Association between gestational diabetes mellitus and depression in parents: A retrospective cohort study. *Clinical Epidemiology* 2018; **10**:1827-1838.

50. Ragland D, Payakachat N, Hays EB, Banken J, Dajani NK, Ott RE. Depression and diabetes: Establishing the pharmacist's role in detecting comorbidity in pregnant women. *Journal of the American Pharmacists Association: JAPhA* 2010; **50**:195-199.

51. Raisanen S, Lehto SM, Nielsen HS, Gissler M, Kramer MR, Heinonen S. Risk factors for and perinatal outcomes of major depression during pregnancy: a population-based analysis during 2002-2010 in Finland. *BMJ Open* 2014; **4**:e004883.

52. Rumbold AR, Crowther CA. Women's experiences of being screened for gestational diabetes mellitus. *Australian & New Zealand Journal of Obstetrics & Gynaecology* 2002; **42**:131-137.

53. Ruohomäki A, Toffol E, Upadhyaya S, Keski-Nisula L, Pekkanen J, Lampi J*, et al.* The association between gestational diabetes mellitus and postpartum depressive symptomatology: A prospective cohort study. *Journal of Affective Disorders* 2018; **241**:263-268.

54. Silverman ME, Reichenberg A, Sandin S, Savitz DA, Cnattingius S, Lichtenstein P*, et al.* The risk factors for postpartum depression: A population-based study. *Depression and Anxiety* 2017; **34**:178-187.

55. Song XF, Liu YJ, Ni CH, Xu ZR, Wang WH, Liu YL. Investigation of depressive symptoms and analysis of related factors in patients with gestational diabetes mellitus. *Chinese Journal of Clinical Rehabilitation* 2004; **8**:6559-6561.

56. Sundaram S, Harman JS, Cook RL. Maternal Morbidities and Postpartum Depression: An Analysis Using the 2007 and 2008 Pregnancy Risk Assessment Monitoring System. *Women's Health Issues* 2014; **24**:e381-e388.

57. Varela P, Spyropoulou AC, Kalogerakis Z, Vousoura E, Moraitou M, Zervas IM. Association between gestational diabetes and perinatal depressive symptoms: Evidence from a greek cohort study. *Primary Health Care Research and Development* 2017; **18**:441-447.

58. Walmer R, Huynh J, Wenger J, Ankers E, Mantha AB, Ecker J*, et al.* Mental Health Disorders Subsequent to Gestational Diabetes Mellitus Differ by Race/Ethnicity. *Depression & Anxiety* 2015; **32**:774-782.

59. Whiteman VE, Ashley Cain M, Salihu HM, Salemi JL, Mejia De Grubb MC, Zoorob RJ*, et al.* Additive effects of Pre-pregnancy body mass index and gestational diabetes on health outcomes and costs. *Obesity* 2015; **23**:2299-2308.

60. Wilson BL, Dyer JM, Latendresse G, Wong B, Baksh L. Exploring the Psychosocial Predictors of Gestational Diabetes and Birth Weight. *JOGNN - Journal of Obstetric, Gynecologic, & Neonatal Nursing* 2015; **44**:760-771.

61. Youn H, Lee S, Han SW, Kim LY, Lee T-S, Oh M-J*, et al.* Obstetric risk factors for depression during the postpartum period in South Korea: a nationwide study. *Journal of Psychosomatic Research* 2017; **102**:15-20.

62. Zwolinska-Kloc M, Zabel M, Czajkowski K, Ostasz-Wazny J, Kokoszka A. Relations between gestational diabetes and postpartum depressive disorders and symptoms. *Archives of Psychiatry and Psychotherapy* 2017; **19**:43-46.

**Table S1 Risk of bias assessment tool**

| Domain | Score (0- high risk of bias, 1- medium risk of bias, 2- low risk of bias) | Comments |
| --- | --- | --- |
| **STUDY DESIGN Total= /2** | | |
| **Suitability of study design for review question**   1. unable to ascertain design of study 2. case control, retrospective cohort or cross-sectional study 3. prospective cohort, interventional or population-level study |  |  |
| **SELECTION BIAS Total= /4** | | |
| **Representativeness of sample**  How were participants recruited? What was the sampling frame?  Could there be any bias from inclusion or exclusion criteria?   1. selection criteria exclude participants of possible interest to the study question or information about the sampling process is not provided 2. some exclusion criteria which could limit the population being studied 3. general population/broad sampling frame |  |  |
| **Participation rates**   1. less than 60% agreed to participate or not reported 2. 60-79% agreement 3. 80-100% agreement or N/A for population-based data |  |  |
| **ATTRITION BIAS Total= /2** | | |
| **Loss to follow up/drop out during the study**  What proportion of those originally recruited completed the study?   1. less than 60% or not reported 2. 60-79% completed 3. 80-100% completed or the study was of a design as to make this N/A (e.g. retrospective cohort) |  |  |
| **MEASUREMENT BIAS Total= /4** | | |
| **Measure of gestational diabetes**  Was this by self-report or clinical diagnosis?   1. no description given or for population-level data, no diagnostic criteria/data extraction algorithm is provided 2. self-report or criteria for diagnosis not specified 3. clinical diagnosis and diagnostic criteria are specified |  |  |
| **Measure of mental disorder**  Was this by clinical interview or screening tool? Is the screening tool validated?   1. no description given, screening tool is not validated or for population-level data or no diagnostic criteria/data extraction algorithm is provided 2. well validated screening tool 3. diagnostic measure |  |  |
| **STATISTICAL ANALYSIS AND RESULTS Total= /4** | | |
| **Confounders**  What proportion of important confounders (ethnicity, BMI, age, socioeconomic status and previous history of mental illness) were controlled for in the design or analysis?   1. less than 60% or unable to ascertain 2. 60-79% 3. 80-100% |  |  |
| **Strengths and weaknesses of the study**  Were they discussed?   1. not at all 2. to some extent 3. fully explored |  |  |
| **FUNDING SOURCE AND CONFLICTS OF INTEREST Total= /4** | | |
| **Funding source**   1. no mention of funding source or source clearly biased the results 2. source of funding may have biased the results 3. source of funding unlikely to have biased the results |  |  |
| **Conflicts of interest**   1. conflicts of interest not declared or clearly biased the results 2. possible conflicts of interest 3. no conflicts of interest |  |  |

**Table S2 Study characteristics, prevalence and odds ratios grouped by mental disorder and time period**

| **Author and year** | **Country** | **Study design and sample size** | **GDM measurement** | **Mental disorder measurement** | **Inclusion criteria** | **Exclusion criteria** | **Odds ratios and/or prevalence** | **Risk of bias** |
| --- | --- | --- | --- | --- | --- | --- | --- | --- |
| **Antenatal depression following GDM diagnosis (GDM as exposure, mental disorder as outcome)** | | | | | | | | |
| **Studies in meta-analysis of odds ratios and prevalence** | | | | | | | | |
| Besser et al, 2007 | Israel | Prospective cohort, N=209 women  GDM- 100 | 50g OGTT at 24-28 weeks.  Two abnormal glucose tolerance tests (at 1 and 3 hours) were designated as GDM. Authors do not specify what these abnormal values are. | CES-D ≥16 in third trimester (after GDM diagnosis- confirmed by contacting study author). | First time mothers, naturally conceived from 10 Well Baby Clinics serving urban lower-middle  class neighborhoods | Prior history of mental or physical illness, including T1 and T2DM, neonatal APGAR <8, miscarriage. | Depression prevalence  GDM- 29/100=29%  No GDM-  35/109=32%  *Calculated from above prevalence figures:*  Unadjusted OR 0.86 (0.48,1.56) | Low to moderate risk of bias  Selection bias  Participation rates: 2  Representativeness: 1  Measurement bias  GDM: 1  Mental disorder: 1  Confounding: 1 |
| Chazotte et al, 1995 | USA | Cross-sectional, N=90 (minus 30 premature controls)=60 women  GDM- 30 | Not specified. | CES-D ≥16 at 34-36 weeks gestation. | Women visiting the prenatal clinic at 34-36 weeks gestation. 30 women in control group- women with uncomplicated low-risk pregnancies, 30 women with GDM and 30 women at risk for preterm delivery. | Not specified. | Depression prevalence  GDM- 17/30=56.7%  Healthy control group- 10/30=33.3%  *Calculated from figures above:*  Unadjusted OR 2.62 (0.92,7.46) | High risk of bias  Selection bias  Participation rates: 0  Representativeness: 0  Measurement bias  GDM: 0  Mental disorder: 1  Confounding: 0 |
| Keskin et al, 2015 | Turkey | Cross-sectional, N=89 women  GDM- 44 | 75g 2h OGTT: plasma glucose during fasting ≥ 92 mg/dL (5.1 mmol/L) or at 1 h ≥ 180 mg/dL (10.0 mmol/L) or at 2 h ≥ 153 mg/dL (8.5 mmol/L). | Antepartum BDI (unknown what version) ≥17 after GDM diagnosis. | Women treated at Istanbul  University, Cerrahpasa Medical Faculty. | Pregestational diabetes, on medication affecting cognitive function, including  corticosteroids, anti-depressants or anti-epileptics. Additionally subjects suffering from any chronic  metabolic, endocrine, inflammatory or infectious diseases, cancer  and subjects who had drug or alcohol dependency, history of  major brain abnormalities (e. g. tumours, hydrocephaly), epilepsy,  Parkinson’s disease or stroke. | Depression prevalence  GDM- 9/44=20.5%  No GDM- 8/45=17.8%  *Calculated from above figures:*  Unadjusted OR 1.19 (0.41,3.43) | High risk of bias  Selection bias  Participation rates: 0  Representativeness: 1  Measurement bias  GDM: 2  Mental disorder: 1  Confounding: 1 |
| Rumbold and Crowther, 2002 | Australia | Prospective cohort, N=209 women  GDM- 25 | 75 g OGTT then diagnosis made according to WHO criteria. | EPDS ≥12 late in third trimester after GDM diagnosis. | Any English-speaking pregnant women  aged ≥18 attending Adelaide Women and Children’s Hospital for antenatal  care who had either been screened or would later  be screened for GDM. | Women with pre-existing diabetes. | Depression prevalence  GDM- 4/21=19%  No GDM- 23/124=  18.5%  *Calculated from above prevalence figures:*  Unadjusted OR 1.03 (0.32,3.36) | High risk of bias  Selection bias  Participation rates: 0  Representativeness: 1  Measurement bias  GDM: 2  Mental disorder: 1  Confounding: 1 |
| Song et al, 2004 | China | Prospective cohort, N=104 women  GDM- 50 | 75g OGTT  Fasting blood glucose ≥ 6.1 mmol/L or 2-hour plasma glucose ≥ 7.8 mmol/L. | SDS (Zung Self-rating depression scale) during pregnancy ≥41; following GDM diagnosis. | Research subjects were selected among pregnant women at the 306^th^ hospital of the People’s Liberation Army on 01/08/03. No other criteria specified. | Not specified. | Depression prevalence GDM- 11/50=22%  No GDM ‘healthy pregnant women’- 4/54=7.4%  *Calculated from above figures:*  Unadjusted OR 3.53 (1.04,11.93) | High risk of bias  Selection bias  Participation rates: 0  Representativeness: 0  Measurement bias  GDM: 2  Mental disorder: 1  Confounding: 0 |
| Varela et al, 2017 | Greece | Prospective cohort, N=117 women  GDM- 17 | Self-report  (confirmed by contacting study author). Diagnostic criteria unknown. | EPDS ≥13 32-35 weeks gestation. | Participants recruited from practice of  collaborating obstetricians in Athens.  Women in their third trimester of pregnancy of  Greek origin or fluent in Greek language, who were  provided with a detailed description of the study  procedures and signed a written informed consent. | Active psychotic symptoms, organic brain  pathology and intellectual disability. Confirmed with study author that T1 and T2DM excluded. | Depression prevalence  GDM-  3/17=17.6%  No GDM-  11/100=11%  Unadjusted OR 1.73 (0.43,7) | High risk of bias  Selection bias  Participation rates: 0  Representativeness: 0  Measurement bias  GDM: 0  Mental disorder: 1  Confounding: 1 |
| **Studies in meta-analysis of prevalence only** | | | | | | | | |
| Bublitz et al, 2017 | USA | Cross-sectional, N=24 women  GDM- 24 | ‘Based on the 1- or 3-h  glucose tolerance test’. | PHQ-9 ≥10 post GDM diagnosis during pregnancy. | Women diagnosed with GDM  were recruited at the time of a clinical visit to  the nurse educator or the nutritionist at a large hospital-based practice  for an evaluation of the association of sleep disturbances with HPA axis function and insulin resistance. | Not specified. | Depression prevalence  GDM- 3/24= 12.5% | High risk of bias  Selection bias  Participation rates: 1  Representativeness: 0  Measurement bias  GDM: 1  Mental disorder: 1  Confounding: 1 |
| Ragland et al, 2010 | USA | Cross-sectional, N=50 women  GDM- 22 | Unknown diagnostic criteria. | BDI version II ≥14 during pregnancy (following GDM diagnosis) | Cross-sectional convenience sample of pregnant women at University Women’s Clinic (UWC) for low-income patients in Little Rock, Arkansas Jun-Aug 2007. | Not specified. | Depression prevalence  GDM- 9/22=40.9%  No GDM- unknown | High risk of bias  Selection bias  Participation rates: 0  Representativeness: 0  Measurement bias  GDM: 0  Mental disorder: 1  Confounding: 1 |
| **Antenatal depression (GDM and mental disorder measured at the same time)** | | | | | | | | |
| **Studies in meta-analysis of odds ratios and prevalence** | | | | | | | | |
| Bisson et al, 2014 | Canada | Case-control study, N=52 women  GDM- 26 | 75g OGTT at 24–28 weeks gestation.  Diagnosis based on 2008 Canadian Diabetes  Association criteria for 75g OGTT and required  two or more abnormal plasma glucose levels: (fasting  ≥95.5 mg/dL, 1-hour ≥191.0 mg/dL  and 2-hour ≥160.4 mg/dL). | EPDS ≥10 at time of study enrolment; mean was 30 weeks gestation. | 18 years or older, singleton pregnancy and  planned delivery at one of the study centres. | Diagnosis of sleep apnea, T1 or T2DM or chronic hypertension before pregnancy  or diagnosed before 20 weeks gestation,  uncontrolled thyroid dysfunction or pre-pregnancy  BMI 35 or greater. | Depression prevalence  GDM-6/26=23.1%  No GDM-  0/26=0%  *Calculated from above prevalence figures, using*  *Haldane-Anscombe correction for zero count:*  Unadjusted OR (GDM as exposure) 16.8 (0.89,315.89) | High risk of bias  Selection bias  Participation rates: 0  Representativeness: 1  Measurement bias  GDM: 2  Mental disorder: 1  Confounding: 1 |
| Byrn and Penckofer, 2015 | USA | Cross-sectional, N=135 women  GDM- 65 | ‘Had medical data to verify GDM status’ but diagnostic criteria not specified. | EPDS ≥12 at 24-40 weeks gestation. | Women who received prenatal care at the research sites,  24-40 weeks gestation, spoke and  read English, had medical data to verify GDM diagnosis,  and were older than age 18. | Women <18, women with T1 or T2DM. | Depression prevalence  GDM- 13/65=  20%  No GDM- 9/70=13%  *Calculated from figures above:*  Unadjusted OR (GDM as exposure) 1.69 (0.67,4.28) | Low to moderate risk of bias  Selection bias  Participation rates: 1  Representativeness: 1  Measurement bias  GDM: 1  Mental disorder: 1  Confounding: 1 |
| Huang et al, 2015 | USA | Prospective cohort (Project Viva), N=2112 women  GDM- 130 | 50g 1 hour non-fasting glucose challenge test (GCT) and if >140 mg/dL, then 100g 3 hour fasting OGTT. GDM if ≥2 of the following:  >95 mg/dL at baseline, >180 mg/dL  at 1 h, >155 mg/dL at 2 h, and >140 mg/dL at 3 h,  according to ADA criteria. | EPDS ≥13 at time of diagnosis. | ≤22 gestational weeks at  enrolment, able to complete questionnaires and interviews  in English and had a singleton pregnancy (between 1999 and 2002). | Not specified. | Prevalence depression  GDM-12.7%  No GDM- 8.8%  Adjusted OR (GDM as exposure) 1.69 (0.88,3.23) *adjusted for age- *used in meta-analysis.*  Adjusted OR 1.47 (0.74,2.93) *adjusted for age, ethnicity, education, place of birth, parity, marital status, household income, pre-pregnancy BMI and pre-pregnancy*  *physical activity.* | High risk of bias  Selection bias  Participation rates: 0  Representativeness: 0  Measurement bias  GDM: 2  Mental disorder: 1  Confounding: 2 |
| Larrabure-Torrealva et al, 2018 | Peru | Cross-sectional (STEM-GDM), N= 1300 women  GDM- 205 | IADPSG criteria so fasting plasma glucose ≥5.1 mmol/l, 1 hour post glucose load ≥10 mmol/l and/or 2 hour post glucose load ≥8.5 mmol/L. | PHQ-9 ≥10 at 24-28 weeks gestation. | Attending prenatal care clinic at the Instituto Nacional Materno Perinatal (INMP) in Lima. At least 18 years of age, gestational age 24-28 weeks and can speak, read and write Spanish. | Planned to deliver at another hospital or location, date of last menstrual period not certain and not confirmed by ultrasound exam performed prior to 24 weeks gestation, unable to complete OGTT, multiple pregnancy, previous diagnosis of diabetes requiring treatment with medication before the pregnancy or currently receiving medical treatment for chronic diseases. | Depression prevalence  GDM- 32/205=  15.6%  No GDM- 106/1095=  9.7%  Unadjusted OR (GDM as exposure) 1.52 (1.09,2.12)  Adjusted OR  *Adjusted for age and family history of DM only*  1.53  (1.09,2.14)  Adjusted OR  1.54 (1.09,2.17)  *Adjusted for maternal age, ethnicity, marital status, maternal education, paternal education, mid-pregnancy BMI, family history of diabetes, perceived health during pregnancy, and difficulty paying for the basics.* | High risk of bias  Selection bias  Participation rates: 0  Representativeness: 1  Measurement bias  GDM: 2  Mental disorder: 1  Confounding: 2 |
| Mautner et al, 2009 | Austria | Prospective cohort, N=40 (29 controls and 11 with GDM)  GDM- 11 | From medical records (clarified with study author). Diagnostic criteria unknown. | EPDS ≥10 at 24-37 weeks gestation. | Jun 06-Aug 07  pregnant women attending the obstetric clinic  at a public hospital, with  an intact pregnancy between 24-37 weeks  gestation, sufficient German language skills. | Complications during early pregnancy (before 24 weeks gestation), including pregnancy loss. | Depression prevalence  GDM- 5/11=  45.5%  No GDM- 6/29=20.7%  Unadjusted OR (GDM as exposure) 3.19 (0.72,14.15)  (raw data provided by study author) | High risk of bias  Selection bias  Participation rates: 2  Representativeness: 1  Measurement bias  GDM: 0  Mental disorder: 1  Confounding: 0 |
| Natasha et al, 2018 | Bangladesh | Cross-sectional, N=748 women  GDM- 382 | WHO and ACOG criteria. Plasma Glucose ≥7.0 (WHO) or ≥5.3 mmol/L at  fasting and ≥8.6 mmol/L at 2 h post 75g OGTT  (ACOG). | MADRS at 24-28 weeks gestation (around the time of GDM diagnosis) ≥13. | Aug 11-Sep 12 at Bangladesh Institute of  Research and Rehabilitation in Diabetes, Endocrine and  Metabolic Disorders (BIRDEM). | >28 weeks gestation, diagnosed diabetes  prior to pregnancy, twin pregnancy, previous GDM, complications due to medical disorder, depression prior to pregnancy or subjects unwilling  to participate. 14 cases of fetal death also excluded. | Depression prevalence  GDM-  99/382=  25.9%  No GDM-  38/366=  10.4%  Unadjusted OR (GDM as exposure) 3.02 (2.01,4.53) | Low to moderate risk of bias  Selection bias  Participation rates: 2  Representativeness: 1  Measurement bias  GDM: 2  Mental disorder: 1  Confounding: 1 |
| **Studies in meta-analysis of prevalence only** | | | | | | | | |
| Dame et al, 2017 | Brazil | Cross-sectional (LINDA-Brazil), N=820 women  GDM- 820 | Self-report and confirmed by medical records (diagnosis made according  to diagnostic criteria used at each centre. Criteria  based on two-step approach (fasting  plasma glucose test followed by OGTT). Initially,  diagnoses were often made using a single elevated two-hour  plasma glucose test. However, more recently, the criteria  developed by the IADPSG  have become more commonly used. | EPDS ≥12 in third trimester. | Receiving care at tertiary prenatal care facilities  within Brazilian National Health System in 2014 and  2015 in three cities: Porto Alegre, Fortaleza, and Pelotas. Recruited as part of RCT. 18 years or older,  with GDM and living within easily  accessible distance to the trial sites. | Not specified. | Depression prevalence  GDM- 31% | High risk of bias  Selection bias  Participation rates: 0  Representativeness: 0  Measurement bias  GDM: 2  Mental disorder: 1  Confounding: 1 |
| Daniells et al, 2003 | Australia | Prospective cohort, N=100  GDM- 50 | ADIPS criteria- fasting glucose ≥5.5 and/or 2-h glucose is ≥8.0 mmol/l. | MHI-5 ≥16 at 30 weeks gestation. | All women attending the Diabetes  Center eligible  if they had GDM, singleton  pregnancy, not previously diagnosed  with GDM, tested after 26  weeks gestation and seen in clinic both within 1 week of diagnosis  and before 32 weeks gestation. All  women had to be able to read and write  English to give informed consent and had  to be willing to follow the study protocol. | Not specified. | Depression prevalence  GDM- 15/50=  30%  No GDM- 6/50=12%  *OR not used for meta-analysis as unclear if T1 and T2DM excluded:*  Unadjusted OR (GDM as exposure) 3.14 (1.1,8.94) | High risk of bias  Selection bias  Participation rates: 1  Representativeness: 0  Measurement bias  GDM: 2  Mental disorder: 1  Confounding: 0 |
| Ghaffar et al, 2016 | Pakistan | Cross-sectional, N=230 women  GDM- 108 | OGTT (unknown load and how many hours post load) >7 mmol/L. | EPDS ≥11 between 13 and 40 weeks. | Pregnant females 16-40 years, with parity <5, >12 weeks gestation presenting with obstetrics risk: pregnancy induced hypertension (BP>140/90mmHg), preeclampsia (PIH with protein urea +1 on dipstick) or eclampsia (convulsions with or without preeclampsia), cervical incompetence, preterm labour, pre mature rupture of membranes (on clinical evaluation), gestational diabetes (GTT>7mmol/L), multiple pregnancy (on USG) or placenta previa (on USG). | Cognitive impairment, not expected to remain hospitalized for more than 72 hours, pre-pregnancy hypertension, diabetes, deranged LFTs or impaired renal function. | Depression prevalence  GDM- 61/108=  56.5%  *Only prevalence used as control (non GDM population is a high-risk pregnancy population so not a valid comparison).* | High risk of bias  Selection bias  Participation rates: 0  Representativeness: 1  Measurement bias  GDM: 1  Mental disorder: 1  Confounding: 0 |
| Hassan et al, 2017 | Iraq | Cross-sectional, N=100  GDM- 50 | 75g OGTT using ADA criteria  Fasting glucose level ≥5.1 and/or 2-h glucose level ≥8.0. | BDI ≥ 20 (moderate to severe depression) (unknown what version of BDI was used) at 24-36 weeks gestation. | Jul-Aug 2015; pregnant women attending two primary health centres for antenatal care. | Not specified. | Depression prevalence  GDM- 43/50=  86%  No GDM- 29/50=58%  *Calculated from prevalence figures above.*  *Not used for meta-analysis as unknown if excluded pregestational diabetes-*  Unadjusted OR (GDM as exposure) 4.45(1.68,11.81) | High risk of bias  Selection bias  Participation rates: 0  Representativeness: 0  Measurement bias  GDM: 2  Mental disorder: 1  Confounding: 1 |
| **Studies not in meta-analysis** | | | | | | | | |
| Egan et al, 2017 | Ireland | Cross-sectional, N=218 (minus 32 with T1DM)= 186 women  GDM- 78 | Unknown source of diagnosis or diagnostic criteria. | DASS 21 item measure of anxiety, depression and stress during pregnancy.  Depression score ≥14. | Participants were recruited from antenatal clinics at a tertiary hospital; no other detail given. | Not specified. | Depression prevalence  GDM- 28/78=  35.9%  No GDM- 27/108=25%  *Calculated from prevalence figures above.*  Unadjusted OR (GDM as exposure) 1.68 (0.89,3.17) | High risk of bias  Selection bias  Participation rates: 0  Representativeness: 0  Measurement bias  GDM: 0  Mental disorder: 1  Confounding: 0 |
| Ferrara et al, 2014 | USA | RCT baseline (GEM study), N=2280 women  GDM- 2280 | 1-h OGTT to screen for GDM. If the screening test is abnormal, a diagnostic 100-g, 3-h OGTT is performed. GDM is diagnosed if 2 or more of the 4 plasma glucose values obtained meet or exceed the plasma glucose thresholds defined by Carpenter and Coustan. | PHQ-8 or PHQ-9 ≥10 at baseline (after GDM diagnosis) or diagnoses of depression or use of antidepressant medications (from searching the electronic health record) during pregnancy. | Women delivering at an integrated health care delivery system, aged 18 or older and diagnosed with GDM between March 11- March 12. | Women with no telephone contact with the Perinatal Center during pregnancy, missing data on pregravid BMI or neonatal loss. | Depression  prevalence  GDM- 443/2163=  20.5% | Low to moderate risk of bias  Selection bias  Participation rates: 2  Representativeness: 1  Measurement bias  GDM: 2  Mental disorder: 1  Confounding: 1 |
| Gemeay et al, 2015 | Saudi Arabia | Cross-sectional, N=100 women (56 with T1 and T2DM)  GDM-44 | Source of diagnosis and diagnostic criteria not specified. | BDI-I ≥ 20 (moderate to severe depression) during pregnancy. | 100 male and female Saudi diabetic patients aged 20-65 years old interviewed between Mar-Jun 14 (convenience sample). | Diabetic complications or psychiatric disorders. | Depression prevalence  GDM- 24/44=  54.5%  No GDM- unknown | High risk of bias  Selection bias  Participation rates: 0  Representativeness: 1  Measurement bias  GDM: 0  Mental disorder: 1  Confounding: 0 |
| Hermon et al, 2018 | Israel | Prospective cohort, N=279 women (minus T1 and T2DM)=273  GDM- 26 | GDM diagnostic criteria not specified. Maternal questionnaires filled upon recruitment containing information regarding maternal background health, obstetrical history and pregnancy course or the computerized perinatal medical files containing all data surrounding the index delivery following the hospitalisation. | EPDS during pregnancy ≥10. | Pregnant women at ≥ 24 weeks gestation of all ages and ethnicities hospitalised at high-risk pregnancy department at the Soroka University Medical Center (SUMC) for any reason.  All hospitalised women were approached regardless of hospitalisation length or indication. | Illiterate women and patients who did not provide an oral and written consent to participate in the study. | Depression prevalence  GDM- 7/26=  26.9%  No GDM (excluding women with pre-gestational)- 71/247=  28.7%  Unadjusted OR (GDM as exposure) 0.91 (0.37,2.27) | High risk of bias  Selection bias  Participation rates: 0  Representativeness: 1  Measurement bias  GDM: 1  Mental disorder: 1  Confounding: 1 |
| Katon et al, 2011 | USA | Cross-sectional, N=2398 women (-226 T1 and T2DM)=2172 women  GDM- 425 | ICD-9 GDM (648.8) from linked medical records. | PHQ-9 ≥10 or current antidepressant use in second or third trimester. | All women receiving prenatal care between Jan 04-Jan 09 at University of Washington Medical Center  completing at least one clinical questionnaire in either the  second or third trimester. | < 15 years at the time of delivery and inability to complete  the clinical questionnaire because of mental incapacitation or  language difficulties (i.e., no interpreter available). | Depression prevalence  GDM- 8.5%  *Numerator not provided in paper but with denominator of 425, 8.5%=36/425 so 36 used as n for purposes of prevalence meta-analysis.*  No GDM or pregestational diabetes- 8.2%  Unadjusted OR (with GDM as exposure) 0.94 (0.68,1.29)  Adjusted OR 0.95 (0.68,1.33)  *Adjusted f**or maternal age, marital status, ethnicity, education, employment, chronic medical conditions, prior pregnancy, gestational week at depression screen, prior pregnancy complication.* | Low to moderate risk of bias  Selection bias  Participation rates: 1  Representativeness: 1  Measurement bias  GDM: 1  Mental disorder: 1  Confounding: 1 |
| Kumpulainen et al, 2018 | Finland | Prospective cohort (PREDO), N=3234 women, excluding T1 and T2DM= 3215  GDM- 344 | Fasting, 1 or 2 hour plasma  glucose during a 75 g OGTT ⩾5.1, 10.0 or  8.5 mmol/l. Extracted from medical records. | CES-D ≥16 during antepartum (unable to ascertain if pre or post GDM diagnosis). | Pregnant women attending antenatal  clinics at one of the 10 study hospitals in Southern and  Eastern Finland for their first ultrasound screen between 12 + 0 and 13 + 6 weeks + days of gestation from 2005-2009. | Not specified, but T1 and T2DM excluded for purposes of analysis. | Depression prevalence  GDM-  73/344=  21.2%  No GDM-  600/2871=  20.9%  Unadjusted OR (GDM as exposure) 1.02 (0.78,1.34)  (raw data provided by study author) | Low to moderate risk of bias  Selection bias  Participation rates: 1  Representativeness: 1  Measurement bias  GDM: 2  Mental disorder: 1  Confounding: 2 |
| Manoudi et al, 2012 | Morocco | Cross-sectional, N=187 patients  GDM- 5 | Not specified; source of diagnosis unknown. | MINI diagnostic interview for DSM-IV depressive disorders during pregnancy. | Opportunistic sampling of patients attending the consultation and endocrinology service of CHU Mohammed VI Marrakech. | Not specified. | Depression prevalence  GDM- 2/5=40%  No GDM- unknown | High risk of bias  Selection bias  Participation rates: 2  Representativeness: 0  Measurement bias  GDM: 0  Mental disorder: 2  Confounding: 1 |
| Ng et al, 2014 | Australia | Prospective cohort (EFHL cohort), N=2231 women (data available for 522 excluding T1 and T2DM)  GDM- 39 | From hospital birth record; diagnostic criteria unknown. | Kessler-6 during pregnancy ≥8. | Women who planned to give birth at one of three participating  hospitals were eligible to participate and enrol  their baby in this study. | Pregnant women aged <16 or unable to provide informed consent. | *Data provided by study author.*  Depression prevalence  GDM- 3/20= 15%  No GDM- 105/502= 20.9%  *Calculated from above prevalence figures:*  Unadjusted OR (GDM as exposure) 0.67  (0.19,2.32) | High risk of bias  Selection bias  Participation rates: 0  Representativeness: 1  Measurement bias  GDM: 1  Mental disorder: 1  Confounding: 0 |
| Raisanen et al, 2014 | Finland | Cross-sectional, N=511938 births  GDM- unknown | ICD-10 GDM diagnoses from Hospital Discharge Register (O24.4). | ICD-10 codes F31.3, F31.5 and F32–34 during pregnancy. | All singleton births between 2002 and 2010. | Multiple births. | Prevalence of GDM in women with antenatal depression-  14.5% with no history of depression and 17.6% with history of depression.  Without antenatal depression-  11.2% with no history of depression and 13.4% with history of depression  Unadjusted OR (GDM as exposure) (excluding T1 and T2DM) 1.49 (1.37,1.62)  Adjusted OR 1.29 (1.11,1.50)  *Adjusted for history of depression prior to pregnancy, maternal age, parity, smoking status, marital status, SES, prior miscarriages, prior terminations, IVF, anaemia, pre-existing diabetes, fear of childbirth and fetal sex.* | Low to moderate risk of bias  Selection bias  Participation rates: 2  Representativeness: 2  Measurement bias  GDM: 1  Mental disorder: 2  Confounding: 1 |
| Ruohomaki et al, 2018 | Finland | Prospective cohort (Kuopio Birth cohort), N=1066  GDM- 150 | From hospital diagnosis records.  Finnish current care guidelines.  OGTT 75g with at least one abnormal value- ≥5.3 mmol/l for fasting stage, ≥10.0 at 1 hour and ≥8.6 at 2 hours. | EPDS ≥10 weeks 28-40 gestation. | All women expected to give birth at Kuopio University Hospital. First pregnancy used for analysis. | Previous mental health issues, previous DM (including T1, T2 or previous GDM), multiple pregnancy, missing EPDS in third trimester of pregnancy, missing information on age or BMI. | Unadjusted OR (depression as exposure) 0.89 (0.49,1.64) | High risk of bias  Selection bias  Participation rates: 0  Representativeness: 1  Measurement bias  GDM: 2  Mental disorder: 1  Confounding: 1 |
| Whiteman et al, 2015 | USA | Retrospective cohort, N=1,057,647  GDM- 51,997 | Delivery record included the 648.8 ICD-9-CM code (abnormal glucose tolerance during pregnancy) and did not have any indication of pre-pregnancy diabetes on the delivery record (ICD-9-CM codes 250.0-250.9) or on the birth certificate. | Indicators on the birth certificate or ICD-9-CM codes documented on the delivery discharge record (maternal) or birth discharge record (infant): depression (293.83, 296.2, 296.3, 300.4, 301.12, 309.0, 309.1, 311).  During pregnancy and up to the point of hospital discharge following delivery. | Livebirth singletons of Florida-resident women from 2004 to 2009 without pre-pregnancy diabetes. | Women with any ICD-9-CM code indicative of pre-pregnancy diabetes, those in which the birth certificate indicated GDM not confirmed by an ICD-9-CM code on the delivery record and conflicting information (documenting both GDM and pre-pregnancy diabetes). Multiple births and infants with chromosomal defects, not 20-44 weeks gestation, or with missing maternal BMI were also excluded. | *Data only available stratified by BMI so not used for meta-analysis.*  In normal BMI category, adjusted OR (with GDM as exposure) 1.44 (1.26,1.65) *Adjusted for maternal age, ethnicity, nativity, education, household income, parity, adequacy of prenatal care, infant sex, and tobacco, alcohol, and drug use during pregnancy.* | Low to moderate risk of bias  Selection bias  Participation rates: 2  Representativeness: 2  Measurement bias  GDM: 1  Mental disorder: 2  Confounding: 2 |
| Wilson et al, 2015 | USA | Prospective cohort (PRAMS survey in Utah), N=3655 women  GDM- unknown | Self-report in PRAMS survey. Yes or no to- “During your most recent pregnancy, were you told  by a doctor, nurse, or other health care worker  that you had gestational diabetes (diabetes that  started during this pregnancy)?” | Cumulative depression was operationalised from  participant responses on three depression questions (unclear what these questions were).  A variable was created in which 0 = *history*  *of depression prior to or during pregnancy,*  1 = *depression prior to but not during pregnancy,*  2 = *of depression during pregnancy but not*  *prior to,* and 3 = *depression prior to and during*  *pregnancy.* | Women participating in PRAMS from 2009-2011 in Utah (random sampling) with linked birth certificate data. Questionnaires completed 2 months after birth. | Diabetes prior to pregnancy, multiple gestations, infants  with birth defects and infants who were  no longer alive at the time of the survey. | Depression prevalence  In those with GDM-  12.3%  No GDM- unknown | High risk of bias  Selection bias  Participation rates: 1  Representativeness: 1  Measurement bias  GDM: 1  Mental disorder: 0  Confounding: 1 |
| **Postnatal depression (GDM as exposure, mental disorder as outcome)** | | | | | | | | |
| **Studies in meta-analysis of prevalence and odds ratios** | | | | | | | | |
| Beka et al, 2018 | Canada | Retrospective cohort, N=326,723 pregnancies  GDM-12,140 | Diagnostic codes (24-28 weeks gestation). Canadian diagnostic criteria not given. | ICD9 and 10 codes for affective disorder up to one year postpartum. Defined  mental illness as having at least 1 hospitalisation, outpatient visit  or physician claim for an affective disorder. | Mothers delivering between 01/04/00- 31/03/09 in Alberta. | Multiple births, mothers younger than 18 or older than 54, not Alberta  residents at time of delivery, pre-existing diabetes (type 1 or 2) or missing information concerning  GDM diagnosis. | Depression prevalence  GDM-1595/12140=  13.1%  No GDM-  39405/314583=12.5%  *Calculated from prevalence figures above:*  Unadjusted OR 1.06(1.00,1.11) | Low to moderate risk of bias  Selection bias  Participation rates: 2  Representativeness: 1  Measurement bias  GDM: 1  Mental disorder: 2  Confounding: 2 |
| Besser et al, 2007 | Israel | Prospective cohort, N=209 women  GDM- 100 | 50g OGTT at 24-28 weeks.  Two abnormal glucose tolerance tests (at 1 and 3 hours) were designated as GDM. Authors do not specify what these abnormal values are. | CES-D ≥16 at 8 weeks postpartum. | First time mothers, naturally conceived from 10 Well Baby Clinics serving urban lower-middle  class neighborhoods | Prior history of mental or physical illness, including T1 and T2DM, neonatal APGAR <8, miscarriage. | Depression prevalence  GDM- 47/100=47%  No GDM-  44/109=40.4%  *Calculated from above prevalence figures:*  Unadjusted OR 1.31 (0.76,2.27) | Low to moderate risk of bias  Selection bias  Participation rates: 2  Representativeness: 1  Measurement bias  GDM: 1  Mental disorder: 1  Confounding: 1 |
| Clark et al, 2018 | USA | Case-control study, N=766 women  GDM- 382 | 1 hour OGTT, 75 g load: 7.8 mmol/l  1 hour OGTT, 100 g load: 10 mmol/l  2 hour OGTT,100 g load:  8.45 mmol/l  Fasting glucose: 5.1 mmol/l | Diagnosis of depression recorded in the charts within six months of delivery (any major depressive disorder, atypical depressive disorder, and depressive disorder not otherwise specified, using DSM-IV codes). | Women who had received obstetric care at Stanford University Hospital between 1998 and 2017. | Women with a history of type I or type II diabetes. | Depression prevalence  GDM-  30/382=7.9%  No GDM-  32/384=8.3%  *Calculated from figures above:*  Unadjusted OR 0.94(0.56,1.58) | Low to moderate risk of bias  Selection bias  Participation rates: 2  Representativeness: 1  Measurement bias  GDM: 2  Mental disorder: 2  Confounding: 2 |
| Hinkle et al, 2016 | USA | Prospective cohort,  N=2802 women  GDM- 107 | Medical record review of OGTT.  Carpenter and Coustan criteria of at least two diagnostic  plasma glucose measurements at or above the defined thresholds  (fasting- 5.3mmol/l; 1 hour- 10.0 mmol/l; 2 hour- 8.6mmol/l; 3 hour-  7.8 mmol/l). If OGTT results were not available but hospital  discharge diagnosis indicated treatment of GDM by medication,  the woman was considered as having had GDM. | EPDS ≥10 at 6 weeks postpartum or self-reported antidepressantmedication use in the postpartum. | Women without pre-existing chronic diseases  or medical conditions, including psychiatric disorders or  diabetes before pregnancy, enrolled at 12 US clinical  centres (2009-2013). Women enrolled in gestational  weeks 8-13. | None specified. | Depression prevalence  GDM- 12/81=14.8%  No GDM- 3/81=3.7%  Unadjusted OR 4.52 (1.23,16.69) | High risk of bias  Selection bias  Participation rates: 1  Representativeness: 0  Measurement bias  GDM: 2  Mental disorder: 0  Confounding: 2 |
| Huang et al, 2015 | USA | Prospective cohort (Project Viva), N=2112 women  GDM- 130 | 50g 1 hour non-fasting glucose challenge test (GCT) and if >140 mg/dL, then 100g 3 hour fasting OGTT. GDM if ≥2 of the following:  >95 mg/dL at baseline, >180 mg/dL  at 1 h, >155 mg/dL at 2 h, and >140 mg/dL at 3 h,  according to ADA criteria. | EPDS ≥13 at 6 months postpartum. | ≤22 gestational weeks at  enrollment, able to complete questionnaires and interviews  in English and had a singleton pregnancy (between 1999 and 2002). | Not specified. | Prevalence depression  GDM- 11%  No GDM- 8.3%    Adjusted OR 1.45 (0.71,2.99) *adjusted for age*  Adjusted OR 1.36 (0.64,2.88) *adjusted for age,* *ethnicity, education, place of birth, parity, marital status, household income, pre-pregnancy BMI and pre-pregnancy*  *physical activity.* | High risk of bias  Selection bias  Participation rates: 0  Representativeness: 0  Measurement bias  GDM: 2  Mental disorder: 1  Confounding: 2 |
| Katon et al, 2014 | USA | Retrospective cohort, N=1423 women minus 104 with pre-pregnancy diabetes=1319 women  GDM- 294 | ICD-9 GDM (648.8) from linked medical records. | PHQ-9 ≥10 at 6 weeks postpartum. | All women receiving prenatal care between Jan 04-Jan 09 at University of Washington Medical Center  completing at least one clinical questionnaire in either the  second or third trimester as well as at 6 weeks postpartum follow-up. | < 15 years at the time of delivery and inability to complete  the clinical questionnaire because of mental incapacitation or  language difficulties (i.e., no interpreter available). | Depression prevalence  GDM-16/294=5.4%  No GDM-55/1025=  5.4%  *Calculated from above figures-*  Unadjusted OR 1.02 (0.57,1.8) | Low to moderate risk of bias  Selection bias  Participation rates: 1  Representativeness: 1  Measurement bias  GDM: 1  Mental disorder: 1  Confounding: 2 |
| Kumpulainen et al, 2018 | Finland | Prospective cohort (PREDO), N=3234 women, excluding T1 and T2DM= 3215  GDM- 344 | Fasting, 1 or 2 hour plasma  glucose during a 75 g OGTT ⩾5.1, 10.0 or  8.5 mmol/l. Extracted from medical records. | CES-D ≥16 at 2 and/or 28 weeks postpartum. | Pregnant women attending antenatal  clinics at one of the 10 study hospitals in Southern and  Eastern Finland for their first ultrasound screen between 12 + 0 and 13 + 6 weeks + days of gestation from 2005-2009. | Not specified, but T1 and T2DM excluded for purposes of analysis. | *Data sent by study author.*  Depression prevalence  GDM-  72/344=  20.9%  No GDM-  544/2871=  18.9%  Unadjusted OR 1.13  (0.86,1.49)  (raw data provided by study author) | Low to moderate risk of bias  Selection bias  Participation rates: 1  Representativeness: 1  Measurement bias  GDM: 2  Mental disorder: 1  Confounding: 2 |
| Mautner et al, 2009 | Austria | Prospective cohort, N=40 (29 controls and 11 with GDM)  GDM- 11 | From medical records (clarified with study author). Diagnostic criteria unknown. | EPDS ≥10 at 3-4 months postpartum. | Jun 06-Aug 07  pregnant women attending the obstetric clinic  at a public hospital, with  an intact pregnancy between 24-37 weeks  gestation, sufficient German language skills. | Complications during early pregnancy (before 24 weeks gestation), including pregnancy loss. | Depression prevalence  GDM-3/11=  27.3%  No GDM-  5/29=17.2%  Unadjusted OR 1.8 (0.35,9.28)  (raw data provided by study author) | High risk of bias  Selection bias  Participation rates: 2  Representativeness: 1  Measurement bias  GDM: 0  Mental disorder: 1  Confounding: 0 |
| Natasha et al, 2018 | Bangladesh | Cross-sectional, N=734 women  GDM- 374 | WHO and ACOG criteria. Plasma Glucose ≥7.0 (WHO) or ≥5.3 mmol/L at  fasting and ≥8.6 mmol/L at 2 h post 75g OGTT  (ACOG). | MADRS ≥13 within one week following delivery. | Aug 11-Sep 12 at Bangladesh Institute of  Research and Rehabilitation in Diabetes, Endocrine and  Metabolic Disorders (BIRDEM). | >28 weeks gestation, diagnosed diabetes  prior to pregnancy, twin pregnancy, previous GDM, complications due to medical disorder, depression prior to pregnancy or subjects unwilling  to participate. 14 cases of fetal death also excluded. | Depression prevalence  GDM-  48/374=  12.8%  No GDM-  15/360=  4.2%  Unadjusted OR 3.39 (1.86,6.17) | Low to moderate risk of bias  Selection bias  Participation rates: 2  Representativeness: 1  Measurement bias  GDM: 2  Mental disorder: 1  Confounding: 1 |
| Nehbandani et al, 2016 | Iran | Prospective cohort, N=262 women  GDM-105 | At least one abnormal result on 75g 2 hour OGTT. Abnormal results include: fasting blood glucose level ≥92 mg/dL, 1 hour after consuming glucose, a blood glucose level that is ≥180 mg/dL, 2 hours after consuming glucose, a blood glucose level that is ≥153 mg/dL. | EPDS ≥12 at 4-6 weeks postpartum. | Being an Iranian citizen, resident in Zabol, age 20-45, singleton pregnancy, full-term, wanted pregnancy, no history of infertility or stillbirth, no adverse incidents in the past year (e.g. loss of significant other, accidents or burglary), no history of postpartum or lifetime depression, no other pregnancy complications including preeclampsia, thyroid problems and anaemia, no antenatal depression at the sampling stage (assessed using BDI) and no known acute or chronic disease in the pregnant women or infant. | Death of a significant other during the study, stillbirth or hospitalisation of the neonate due to illness. | Depression prevalence  GDM- 36/105=  34.3%  No GDM- 30/157=  19.1%  *Calculated from above prevalence figures (differs from 1.79 (1.37,2.2) cited in paper- authors e-mailed for clarification but no response received.*  Unadjusted OR 2.21 (1.25,3.89) | High risk of bias  Selection bias  Participation rates: 0  Representativeness: 2  Measurement bias  GDM: 2  Mental disorder: 1  Confounding: 1 |
| Silverman et al, 2017 | Sweden | Prospective cohort, N=707,701 women (but pregestational DM excluded)= 701,404 women  GDM-3289 | From medical birth register. | ICD 9 and 10 depression up to one year postpartum.  This included diagnoses of PPD, as well as major depressive  disorder (single or recurrent), unspecified episodic mood disorder,  or depressive disorder that occurred within the first year postpartum  (ICD-9 296.20, 296.21, 296.22, 296.23, 296.3, 296.31, 296.32, 296.33, 296.34, 296.99, 301.1, 309.0, 311, 311.0,  648.40, 648.42, 648.44  ICD-10 F32, F320, F321, F322, F32.3,a F32.4, F32.8, F32.9, F33, F33.0, F33.1, F33.2, F33.3, F33.4, F33.8, F33.9,  F34.0, F34.1, F34.8, F34.9, F38.0, F38.1, F38.8, F39, F53, F530, F53.1, F53.8, F53.9). | National registers identified a cohort comprising  all Swedish-born women who delivered a live singleton infant between  01/01/97-31/12/08.  Information only on first childbirth  during study period for each woman. | Not specified. | Depression prevalence GDM- 46/3289=1.4%  No GDM (also excluding pregestational DM)- 4273/698115=0.6%  Unadjusted OR *using above figures* 2.3 (1.72,3.09) | High risk of bias  Selection bias  Participation rates: 2  Representativeness: 2  Measurement bias  GDM: 1  Mental disorder: 2  Confounding: 0 |
| Varela et al, 2017 | Greece | Prospective cohort, N=93 women  GDM- 14 | Self-report  (confirmed by contacting study author). Diagnostic criteria unknown. | EPDS ≥13 in the first postpartum week. | Participants recruited from practice of  collaborating obstetricians in Athens.  Women in their third trimester of pregnancy of  Greek origin or fluent in Greek language, who were  provided with a detailed description of the study  procedures and signed a written informed consent. | Active psychotic symptoms, organic brain  pathology and intellectual disability. Confirmed with study author that T1 and T2DM excluded. | Depression prevalence  GDM-  5/14=35.7%  No GDM-  9/79=11.4%  Unadjusted OR 4.32 (1.18,15.78) | High risk of bias  Selection bias  Participation rates: 0  Representativeness: 0  Measurement bias  GDM: 0  Mental disorder: 1  Confounding: 1 |
| Zwolinska-Kloc et al, 2017 | Poland | Case-control, N=70 women  GDM- 35 | Diagnosis by OGTT; time not specified in paper but author confirmed at 24- 28 weeks gestation in response to my e-mail request for further info. Unknown diagnostic criteria. | MINI ICD-10 diagnostic interview 6-7 months postpartum. Also the HADS at 5-8 months gestation, 2 weeks postpartum and 6 weeks postpartum (data not reported and unavailable). | Absence of pre-existing mental disorder on MINI ICD-10 diagnostic interview at enrolment. | T1 and T2DM (not reported; confirmed by contacting study author). | Depression prevalence  GDM: 3/35=8.6%  No GDM: 0/35=0%  *Calculated from above prevalence figures, using*  *Haldane-Anscombe correction for zero count:*  Unadjusted OR 7.65 (0.38,153.76)  Authors report ‘no difference in mean HADS scores between groups’. | High risk of bias  Selection bias  Participation rates: 0  Representativeness: 1  Measurement bias  GDM: 1  Mental disorder: 2  Confounding: 0 |
| **Studies in meta-analysis of odds ratios only** | | | | | | | | |
| Ruohomaki et al, 2018 | Finland | Prospective cohort (Kuopio Birth cohort), N=1066  GDM- 150 | From hospital diagnosis records.  Finnish current care guidelines.  OGTT 75g with at least one abnormal value- ≥5.3 mmol/l for fasting stage, ≥10.0 at 1 hour and ≥8.6 at 2 hours. | EPDS ≥10 at 8 weeks postpartum. | All women expected to give birth at Kuopio University Hospital. First pregnancy used for analysis. | Previous mental health issues, previous DM (including T1, T2 or previous GDM), multiple pregnancy, missing EPDS in third trimester of pregnancy, missing information on age or BMI. | Unadjusted OR 1.84 (1.13,3.00)  Adjusted OR 1.70 (1.00-2.89) *adjusted for age at delivery and first trimester BMI*  Adjusted OR 1.83 (1.08,3.11)  *adjusted as above plus* *smoking before pregnancy, living with partner, nulliparity, gestational age at delivery, C section and NICU admission*  Adjusted OR 2.23 (1.23,4.05)  *adjusted as above in model 2 plus further adjusted for third trimester EPDS scores* | High risk of bias  Selection bias  Participation rates: 0  Representativeness: 1  Measurement bias  GDM: 2  Mental disorder: 1  Confounding: 1 |
| Walmer et al, 2015 | USA | Prospective cohort, N=18,888 pregnancies  GDM- 696 | 1 hour 50g glucose load test (GLT) ≥7.8 and 3 hour 100g OGTT with 2  or more abnormal values according to Carpenter–Coustan criteria. | ICD-9 codes for depression (296.3, 309.0, 309.1, 311, 300.4, 296.2) from electronic medical  records that comprised inpatient and outpatient encounters in the postpartum. | Women presenting to Massachusetts General Hospital (MGH) for prenatal care between 1998 and 2007. Age 18–40,  live births and biochemically confirmed GDM diagnosis. | Women with  a history of mental health disorders prior to delivery or missing self-reported  ethnicity data. Also  prior T1 or T2DM (not reported but confirmed with study author). | Age adjusted OR 1.45 (1.15,1.82)  Complication adjusted OR *(age, pre-eclampsia, pre-term birth)*  1.46 (1.16,1.83)  Multivariable adjusted OR (*age, pre-eclampsia, preterm birth, marital status, years of education*, *baby gender, mode of delivery, primary language spoken, number*  *of fetuses, other labor complications, systolic blood pressure, parity, BMI, weight gain, breast feeding at discharge, and length of*  *follow-up)*  1.29 (0.98,1.70) | Low to moderate risk of bias  Selection bias  Participation rates: 2  Representativeness: 1  Measurement bias  GDM: 2  Mental disorder: 2  Confounding: 1 |
| **Studies in meta-analysis of prevalence only** | | | | | | | | |
| Al Shahrani et al, 2011 | Saudi Arabia | Prospective cohort, N=113 women  GDM-56 | Results of OGTT (75g) but diagnostic criteria not specified. | EPDS ≥12 at 1 week postpartum. | Women giving birth at Abha  Maternity Hospital from 01/01/01-31/03/01. | Not specified. | Depression prevalence  GDM-23/56=41.1%  No GDM-21/57=36.8%  *Calculated from prevalence data given above but not used for meta-analysis as unknown if excluded pregestational diabetes-*  Unadjusted OR 1.19  (0.56,2.55) | High risk of bias  Selection bias  Participation rates: 0  Representativeness: 0  Measurement bias  GDM: 1  Mental disorder: 1  Confounding: 1 |
| Bener et al, 2012 | Qatar | Cross-sectional, N=1379 women  GDM- 94 | Does not specify. | EPDS ≥12 within 6 months of delivery. | Arab women  residing in Qatar at 6 months postpartum attending  12 primary healthcare centres throughout Qatar  from Jan 10- May 11. | Beyond 6 months postpartum, refused  to give consent and/or past psychiatric treatments in medical  records. | Depression prevalence  GDM-  24/94=25.5%  No GDM-  219/1285=  17%  *Not used for meta-analysis as unknown if excluded pregestational diabetes (calculated from above prevalence figures)-*  Unadjusted OR 1.67 (1.03,2.71) | High risk of bias  Selection bias  Participation rates: 2  Representativeness: 0  Measurement bias  GDM: 0  Mental disorder: 1  Confounding: 1 |
| Berger et al, 2015 | USA | Retrospective cohort, N=537 women  GDM- 56 | Diagnostic criteria not specified and source of the diagnosis unknown. | EPDS ≥13 between day 0 and day 4 postpartum. | Women delivering at Lehigh  Valley Health Network (LVHN) in Philadelphia from 01/01/10-  30/06/10, completing the EPDS. | Women who did not complete the EPDS. | In the unselected group *used for prevalence-  Depression prevalence  GDM-3/31=9.7%  No GDM-8/291=2.7%  *Not included in meta-analysis as pregestational DM not excluded from control population-*  Unadjusted OR 3.79 (0.95,15.1)  In the high risk group with a history of mental illness-  Depression prevalence  GDM-6/25=24%  No GDM-27/190=  14.2%  *Not included in meta-analysis as pregestational DM not excluded from control population-*  Unadjusted OR 1.91(0.7,5.2) | High risk of bias  Selection bias  Participation rates: 2  Representativeness: 2  Measurement bias  GDM: 0  Mental disorder: 1  Confounding: 1 |
| Blom et al, 2010 | Netherlands | Prospective cohort (Generation R), N=4941 women  GDM- 32 | From midwife and hospital registries-  diagnosed according to Dutch midwifery and  obstetric guidelines using the following criteria: random  glucose level > 11.1 mmol/l or a glucose level > 7.0 mmol/l  after fasting, in the absence of previously diagnosed  diabetes. | EPDS ≥13 at two months postpartum. | All women living in Rotterdam, the Netherlands,  with an expected delivery date between Apr 02-Jan 06 were eligible for participation. | Not specified. | Depression prevalence  GDM- 4/32=  12.5%  No GDM- 396/4941=  8%  *Calculated from above figures; not used for meta-analysis as unknown if excluded pregestational DM.*  Unadjusted OR 1.64 (0.57,4.7) | Low to moderate risk of bias  Selection bias  Participation rates: 1  Representativeness: 1  Measurement bias  GDM: 2  Mental disorder: 1  Confounding: 1 |
| Farr et al, 2014 | USA | Prospective cohort (PRAMS survey), N=4451 women  GDM- unknown | Self-report GDM. | Self-report postpartum depression. Yes or no to ‘I have felt down, depressed or  sad’, ‘I have felt hopeless’ and ‘I have felt slowed down  physically.’- score ≥6 validated against SCID diagnostic interview. | Women participating in PRAMS in 2009 and 2010 in Illinois and Maryland (random sampling strategy used in PRAMS). To  be included in the sample, questionnaires must have been  completed within 9 months of delivery. | Not specified. | Depression prevalence  GDM- 2.8% (plus 8.1% co-morbid depression and anxiety)= 10.9%  No GDM- 2.6% (plus 6.1% co-morbid depression and anxiety)=  8.7%  Adjusted OR for co-morbid depression and anxiety 1.4 (0.8-2.4)  Adjusted OR for depression 1.0 (0.5-2.0)  *Adjusted for income, pre-pregnany BMI, alcohol, Medicaid coverage, stressful life events in pregnancy, mode of delivery, infant birthweight and gestational age.* | Low to moderate risk of bias  Selection bias  Participation rates: 1  Representativeness: 1  Measurement bias  GDM: 1  Mental disorder: 1  Confounding: 1 |
| Ferrari et al, 2018 | Germany | Cross-sectional, N=173 women  GDM- 173 | GDM within the last year- OGTT and IADPSG criteria. | BDI- I (in 16%) and BDI-II (in 84%) up to one year postpartum. ≥10 for BDI-I and ≥14 for BDI-II ‘mild-moderate’ depression. | GDM diagnosis according to IADPSG criteria. | Alcohol or substance misuse, pre-pregnancy diabetes, (post)menopausal status or chronic diseases requiring systemic medication. CIDI diagnostic interview also used to exclude psychiatric disorders in the first 100 patients. | Depression prevalence  GDM- 22/173=  12.7% | High risk of bias  Selection bias  Participation rates: 0  Representativeness: 0  Measurement bias  GDM: 2  Mental disorder: 1  Confounding: 1 |
| Gunderson et al, 2015 | USA | Prospective cohort (SWIFT), N=1035 women  GDM- 1035  (959 with depression data) | Carpenter-Coustan criteria. | CES-D ≥16 at  6-9 weeks postpartum. | Enrolled between Aug 08-Dec 11, received prenatal care and delivered at Kaiser Permanente Northern California hospitals.  Diagnosis of GDM, live birth at 35 weeks gestation or later, age 20 to 45 years, no history of diabetes, English- or Spanish-speaking, no serious medical conditions and classification of infant feeding as intensive lactation or intensive formula feeding. | Mixed feeding. | Depression prevalence  GDM- 127/959=  13.2% | Low to moderate risk of bias  Selection bias  Participation rates: 1  Representativeness: 2  Measurement bias  GDM: 2  Mental disorder: 1  Confounding: 1 |
| Kim et al, 2005 | USA | Prospective cohort (WISH cohort), N=1445  GDM- 64 | Source of diagnosis from medical record but diagnostic criteria not specified. | Short form CES-D 10 items ≥11 at 8-12 weeks postpartum. | Receiving prenatal care at  one of six San Francisco Bay Area hospitals,  ≥18 years old, speak English, Spanish or Cantonese,  present for prenatal care before 16  weeks gestation and contactable by  telephone. Women were enrolled between May  01-July 02. | Not specified. | Prevalence depression  GDM- 14.1%  *Numerator not provided in paper but with denominator of 64, 14.1%=9/64 so 9 used as n for purposes of prevalence meta-analysis.*  No GDM- unknown | Low to moderate risk of bias  Selection bias  Participation rates: 2  Representativeness: 2  Measurement bias  GDM: 1  Mental disorder: 1  Confounding: 1 |
| Koutra et al, 2018 | Greece | Prospective cohort (Rhea mother-child cohort), N=1037 women  GDM- 76 | Two or  more of the following post 100g 3 hour OGTT  as per Carpenter & Coustan: fasting blood glucose  ≥95 mg/dl; 1-h ≥180 mg/dl; 2-h values ≥155 mg/dl;  and 3-h ≥140 mg/dl. | EPDS ≥13 at 8 weeks postpartum. | Female residents (Greek and immigrants)  who became pregnant during the 12-month period  starting in Feb 07 at  four maternity clinics in Heraklion, Crete.  Women had to have a good understanding  of the Greek language and be older than 16 years of  age. | 7 women with a previous diagnosis of severe psychiatric  disorders (e.g. schizophrenia, bipolar disorder)  and 28 multiple pregnancies were excluded. | Depression prevalence  GDM- 14/76=  18.4%  No GDM- 127/961=  13.2%  *Not used for meta-analysis as unknown if pregestational diabetes excluded-*  Unadjusted  OR 1.46 (0.79,2.71)  Adjusted OR  1.53(0.82,2.87)  *Adjusted for maternal age, education, origin, marital status, working during pregnancy and parity.* | Low to moderate risk of bias  Selection bias  Participation rates: 2  Representativeness: 1  Measurement bias  GDM: 2  Mental disorder: 1  Confounding: 1 |
| Nicklas et al, 2013 | USA | RCT baseline (TEAM GDM), N=71 women  GDM- 71 | Carpenter-Coustan criteria for GDM. | EPDS ≥9 at 4-15 weeks postpartum. | Women planning to deliver or who had delivered at the Brigham and Women’s Hospital  with GDM. Age 18–45, self-reported pre-pregnancy  BMI 18–50, no personal history of T2DM and delivery ≥32 weeks gestation. | Women with a BMI <24 (<22 for Asian participants) or >50 at 6 weeks postpartum. | Depression prevalence  GDM- 24/71=  33.8% | High risk of bias  Selection bias  Participation rates: 0  Representativeness: 1  Measurement bias  GDM: 2  Mental disorder: 1  Confounding: 2 |
| O’Reilly et al, 2016 | Australia | RCT baseline (MAGDA), N=573 women  GDM- 573 | GDM within the last year.  Australasian Diabetes in Pregnancy Society (ADIPS) criteria  at the time of study commencement: fasting plasma glucose (FPG) of ≥5.5 mmol/l  or 2-h glucose of ≥8.0 mmol/l on 75-g OGTT or  glucose challenge test result of ≥11.1 mmol/l. | 3 months postpartum PHQ-9 ≥10 (at RCT baseline). | Women aged >18 with a diagnosis of GDM in their most recent pregnancy. | Pre-existing diabetes, cancer (not in remission), severe mental  Illness, substance abuse, myocardial infarction in the preceding 3 months, difficulty  with English, involvement in another postnatal intervention trial and pregnancy at postnatal  baseline testing or at any point during the 12 months of study involvement. | Depression prevalence  GDM- 59/569=  10.4% | Low to moderate risk of bias  Selection bias  Participation rates: 1  Representativeness: 1  Measurement bias  GDM: 2  Mental disorder: 1  Confounding: 1 |
| Youn et al, 2017 | South Korea | Retrospective cohort (population-based), N=1,269,130 women  GDM- 40,743 | ICD-10 GDM. Diagnostic criteria unknown. | Depression up to one year postpartum ICD-10 codes: F32 and F33. | Database of Korean National Health Insurance Service. Data extracted on women who gave birth between 1/1/10 and 31/12/12. When more than one pregnancy, first pregnancy only was used. | Not specified. | Depression prevalence GDM 1606/40743=3.9%  No GDM: 15,877/1,228,387=1.3%  *Not used for meta-analysis as unclear if T1 and T2DM have been excluded:*  Unadjusted OR (*calculated from above figures*) 3.13 (2.97,3.3)  Adjusted OR 1.04 (0.98,1.11)  *(Adjusted for age, parity, Caesarean delivery, multiple pregnancy, induced labour, pre-eclampsia, preterm* *delivery, placenta previa, placental abruption, uterine artery embolisation, peripartum hysterectomyand previous depression)* | High risk of bias  Selection bias  Participation rates: 2  Representativeness: 2  Measurement bias  GDM: 1  Mental disorder: 2  Confounding: 0 |
| **Studies not in meta-analysis** | | | | | | | | |
| Abdollahi et al, 2014 | Iran | Prospective cohort, N=1546 women  GDM- unknown | Self-report, Iranian diagnostic criteria not reported. | EPDS (Iranian version) ≥13 at either 0-2, >2-8 or >8-12 weeks postpartum. | Pregnant women,  literate in Persian, 32–42 weeks gestation, attending prenatal care at primary health centres of Mazandaran University  of Medical Sciences  from Jan-Jun 2009. | Depression during 32–42 weeks gestation according to EPDS in the study or on medication for psychiatric problems. | *Not used for meta-analysis as unknown if pregestational DM was excluded-*  Adjusted OR 2.93 (95% CI 1.46-5.88)  Unadjusted OR 2.19 (1.26-3.8) | Low to moderate risk of bias  Selection bias  Participation rates: 2  Representativeness: 1  Measurement bias  GDM: 1  Mental disorder: 1  Confounding: 1 |
| Liu and Tronick, 2013 | USA | Prospective cohort (PRAMS survey in NYC), N=3732 women  GDM- unknown | Self-report GDM in PRAMS survey. Yes or no to: ‘‘High blood sugar (diabetes) that  started during this pregnancy’’. | Self-report postnatal depression. Yes or no to: ‘‘Since your new baby was born, has a doctor,  nurse, or other health care worker diagnosed you with  depression?’’ | Women participating in PRAMS from 2004-2007 (random sampling). | Not providing consent. | *Unknown if excluded women with pregestational DM so not used in meta-analysis-*  Adjusted OR 0.8(0.4,1.6)  *Unclear what sociodemographic variables were controlled for.* | High risk of bias  Selection bias  Participation rates: 1  Representativeness: 1  Measurement bias  GDM: 1  Mental disorder: 0  Confounding: 1 |
| Sundaram et al, 2014 | USA | Prospective cohort (PRAMS survey), N=61,733 pregnancies  GDM- 10,691 | Self-report in PRAMS survey.  “Did you  have any of these problems during your most recent pregnancy?” then select:  ‘Gestational diabetes’. | Outcome  Self-report PPD. Yes or no to 1) ‘‘Since your new baby was born, has a doctor,  nurse, or other health care worker diagnosed you with  depression?’’  2) “Since your new baby was born, how often have you felt down,  depressed, or hopeless?”, 3) ‘’Since your new baby was born,  how often have you had little interest or little pleasure in doing  things?”  For the two symptoms questions, adopted a scoring system like the PHQ-2 thus scores ≥3 indicating depression. | Women participating in PRAMS in 2007 and 2008 across 40 states and NYC (random sampling strategy used in PRAMS). | Not specified. | *Not used for meta-analysis as unclear if T1 and T2DM have been excluded:*  Unadjusted OR for depression symptoms 1.13 (0.93,1.30)  Unadjusted OR for depression diagnosis  OR 0.96 (0.64,1.52) | High risk of bias  Selection bias  Participation rates: 1  Representativeness: 1  Measurement bias  GDM: 1  Mental disorder: 0  Confounding: 1 |
| **Antenatal anxiety (GDM and mental disorder measured at the same time)** | | | | | | | | |
| Boggaram et al, 2017 | India | Cross-sectional, N=100 women  GDM- 11 | Does not specify how GDM was measured. Study author informs was ‘diagnosed by trained OB/GYN’. | MINI structured interview during pregnancy (unknown if MINI ICD10 or DSM-IV) for anxiety disorders. | All pregnant women aged >18 attending antenatal clinics. | Unwilling or unable to give informed consent. | Anxiety prevalence (from data provided by study author)  GDM-  3/11=27.3%  No GDM-  9/89=10.1%  *Calculated from prevalence figures above.*  *ORs not used for meta-analysis as pregestational DM not excluded-*  Unadjusted OR (GDM as exposure) 3.33 (0.75,14.87) | High risk of bias  Selection bias  Participation rates: 2  Representativeness: 2  Measurement bias  GDM: 0  Mental disorder: 2  Confounding: 0 |
| Egan et al, 2017 | Ireland | Cross-sectional, N=218 (minus 32 with T1DM)= 186 women  GDM- 78 | Unknown source of diagnosis or diagnostic criteria. | DASS 21 item measure of anxiety, depression and stress during pregnancy.  Anxiety score ≥10. | Participants were recruited from antenatal clinics at a tertiary hospital; no other detail given. | Not specified. | Anxiety prevalence  GDM- 45/78=  57.7%  No GDM- 54/108=50%  *Calculated from prevalence figures above.*  Unadjusted OR (GDM as exposure) 1.36 (0.76,2.45) | High risk of bias  Selection bias  Participation rates: 0  Representativeness: 0  Measurement bias  GDM: 0  Mental disorder: 1  Confounding: 0 |
| Hassan et al, 2017 | Iraq | Cross-sectional, N=100  GDM- 50 | 75g OGTT using ADA criteria  Fasting glucose level is ≥5.1 and/or 2-h glucose level ≥8.0. | Taylor anxiety scale ≥25 (moderate to severe anxiety) at 24-36 weeks gestation. | Jul-Aug 2015; pregnant women attending two primary health centres for antenatal care. | Not specified. | Anxiety prevalence  GDM- 43/50=86%  No GDM (‘glucose tolerant’)- 34/50=68%  *Calculated from prevalence figures above:*  Unadjusted OR (GDM as exposure) 2.89  (1.07,7.82) | High risk of bias  Selection bias  Participation rates: 0  Representativeness: 2  Measurement bias  GDM: 2  Mental disorder: 1  Confounding: 1 |
| **Postnatal anxiety (GDM as exposure, mental disorder as outcome)** | | | | | | | | |
| Beka et al, 2018 | Canada | Retrospective cohort, N=326,723 pregnancies  GDM-12,140 | Diagnostic codes (24-28 weeks gestation). Canadian diagnostic criteria not given. | ICD9 and 10 codes for anxiety disorder up to one year postpartum. Defined  mental illness as having at least 1 hospitalisation, outpatient visit  or physician claim for an anxiety disorder. | Mothers delivering between 01/04/00- 31/03/09 in Alberta. | Multiple births, mothers younger than 18 or older than 54, not Alberta  residents at time of delivery, pre-existing diabetes (type 1 or 2) or missing information concerning  GDM diagnosis. | Anxiety prevalence  GDM-  1772/12140=14.6%  No GDM-  43001/314583= 13.7%  *Calculated from prevalence figures above:*  Unadjusted OR 1.08 (1.03,1.14) | Low to moderate risk of bias  Selection bias  Participation rates: 2  Representativeness: 1  Measurement bias  GDM: 1  Mental disorder: 2  Confounding: 2 |
| Farr et al, 2014 | USA | Prospective cohort (PRAMS survey), N=4451 women  GDM- unknown | Self-report GDM. | Self-report postpartum anxiety. Yes or no to ‘I have felt restless or fidgety’ and ‘I have felt panicky’- score ≥6 validated against SCID diagnostic interview. | Women participating in PRAMS in 2009 and 2010 in Illinois and Maryland (random sampling strategy used in PRAMS). To  be included in the sample, questionnaires must have been  completed within 9 months of delivery. | Not specified. | Anxiety prevalence  GDM- 10.3% (plus 8.1% co-morbid depression and anxiety)= 18.4%  No GDM- 11.5% (plus 6.1% co-morbid depression and anxiety)=  17.6%  Adjusted OR for co-morbid depression and anxiety 1.4 (0.8-2.4)  Adjusted OR for anxiety  1.0 (0.6-1.5)  *Adjusted for income, pre-pregnany BMI, alcohol, Medicaid coverage, stressful life events in pregnancy, mode of delivery, infant birthweight and gestational age.* | Low to moderate risk of bias  Selection bias  Participation rates: 1  Representativeness: 1  Measurement bias  GDM: 1  Mental disorder: 1  Confounding: 1 |
| Walmer et al, 2015 | USA | Prospective cohort, N=18,888 pregnancies  GDM- 696 | 1 hour 50g glucose load test (GLT) ≥7.8 and 3 hour 100g OGTT with 2  or more abnormal values according to Carpenter–Coustan criteria. | ICD-9 codes for anxiety (300.0, 300.00, 300.01, 300.02, 300.09) from electronic medical  records that comprised inpatient and outpatient encounters in the postpartum. | Women presenting to Massachusetts General Hospital (MGH) for prenatal care between 1998 and 2007. Age 18–40,  live births and biochemically confirmed GDM diagnosis. | Women with  a history of mental health disorders prior to delivery or missing self-reported  ethnicity data. Also  prior T1 or T2DM (not reported but confirmed with study author). | Age adjusted OR 1.36 (1.03,1.79)  Complication adjusted OR *(age, pre-eclampsia, pre-term birth)*  1.36 (1.03,1.79)  Multivariable adjusted OR (*age, pre-eclampsia, preterm birth, marital status, years of education*, *baby gender, mode of delivery, primary language spoken, number*  *of fetuses, other labor complications, systolic blood pressure, parity, BMI, weight gain, breast feeding at discharge, and length of*  *follow-up)*  1.14 (0.83,1.57) | Low to moderate risk of bias  Selection bias  Participation rates: 2  Representativeness: 1  Measurement bias  GDM: 2  Mental disorder: 2  Confounding: 1 |

**Table S3Characteristics of studies measuring GDM and mental disorder but data not presented as prevalence or odds ratios**

| **Author and year** | **Country** | **Study design and sample size** | **GDM measurement** | **Mental disorder measurement** | **Inclusion criteria** | **Exclusion criteria** | **Key findings** | **Quality appraisal** |
| --- | --- | --- | --- | --- | --- | --- | --- | --- |
| **Antenatal depression (GDM and mental disorder measured at the same time)** | | | | | | | | |
| Dalfra et al, 2012 | Italy | Prospective cohort, N=245 women minus 30 T1DMs=215  GDM- 176 | 100g OGTT and using Carpenter-Coustan criteria. | CES-D ≥16 in third trimester and at 8 weeks postpartum. | Patients enrolled at  12 Italian diabetes clinics. | Any patients with pre-eclampsia and cases of fetal malformations  or fetal mortality or any other concomitant  diseases. | Mean (SD) CES-D scores at 3rd trimester were 17.0 (8.6) among women  with GDM and 18.0 (8.7) among women without GDM (p=0.52). The severity of depressive symptoms increased from the 3rd trimester to  after delivery in women with GDM (estimated mean difference in CES-D  score (95%CI): 5.7 (4.2,7.3)) but decreased in women without GDM (‑2.7  (-5.9,0.5)); p< 0.0001. | High risk of bias  Selection bias  Participation rates: 0  Representativeness: 1  Measurement bias  GDM: 2  Mental disorder: 1  Confounding: 0 |
| Fiskin et al, 2018 | Turkey | RCT baseline,  N=60 women  GDM- 60 | Unknown source of diagnosis or diagnostic criteria. | DASS 42 item measure of anxiety, depression and stress at 24-28 weeks gestation. | Singleton pregnant women with GDM who were on regular polyclinic follow-up of Istanbul University Medical School, Department of Obstetrics and Gynaecology between June 2015–June 2016.  Between 19 and 35, normal weight (BMI <25kg/m²), whose diabetes was regulated by diet. | Pregnant women with pre-existing diabetes and those who were unable to write and understand Turkish. | DASS depression score mean (SD)  Experimental arm: 4.7 (4.6)  Control arm: 5.3 (5.1) | High risk of bias  Selection bias  Participation rates: 0  Representativeness: 1  Measurement bias  GDM: 0  Mental disorder: 1  Confounding: 1 |
| Levy-Shiff et al, 2002 | Israel | Prospective cohort, N=153 women (minus 53 with pre-gestational DM)=100 women  GDM- 51 | 1 hour 50g OGTT. If >130 mg/dL, then 3 hour 100g OGTT test performed. ‘Two or more abnormal test values’ but does not specify what they are. | BDI in second trimester (unclear what version of the BDI). | Recruited from a high-risk pregnancy clinic in a central hospital. Medical records were screened then women invited to participate. | Not specified. | No significant difference in depression scores during second trimester between  GDM (mean (SD) BDI score 6.70 (4.46)) and controls (6.59 (5.88), p=0.42). | High risk of bias  Selection bias  Participation rates: 2  Representativeness: 0  Measurement bias  GDM: 1  Mental disorder: 1  Confounding: 0 |
| Mak et al, 2018 | China | Prospective cohort, N=1449  GDM- 229 | 75g 1 hour and 2 hour OGTT at 24-28 weeks gestation. IADPSG criteria- fasting serum glucose ≥5.1 mmol/L, 1-h serum glucose ≥10.0 mmol/L, or 2-h serum glucose ≥8.5 mmol/L. | Chinese version of EPDS at 32-37 weeks gestation. | May-Aug 2015 recruited at four maternity hospitals in Chengdu during routine antenatal care. Age 18–40 years, singleton pregnancy. | Without infertility treatment and absence of any severe chronic or infectious diseases. Excluded women with pre-gestational DM from analysis. | Antenatal EPDS  GDM (mean (SD): 9.2 (3.4)  No GDM: 9.3 (3.7)  p=0.772 | Low to moderate risk of bias  Selection bias  Participation rates: 2  Representativeness: 1  Measurement bias  GDM: 2  Mental disorder: 1  Confounding: 1 |
| Pace et al, 2018 | Canada | Retrospective cohort, N=58,400 mothers  GDM-29,200 | ICD-9: 6480 or 6488, ICD-10: O24.8. | ICD-9 and 10 codes: ≥2 outpatient visit diagnoses for depression  within a 2 year time frame or one hospitalisation discharge  diagnosis or hospital admission for self-harm or suicide recorded as a cause of death. | Women aged 20–44 years who, between April 1, 1990,  and December 31, 2007, 1) had a singleton live birth and  2) had ≥2 outpatient physician billing diagnoses for GDM  within 6 months  of delivery and/or a postdelivery hospitalisation discharge  GDM diagnosis. | Pre-gestational DM or who had a history of psychiatric illness (mood and psychotic disorders) in the 1 year prior to 24 weeks gestation. | 24 weeks gestation to delivery:  Unadjusted HR for depression in GDM pregnancy- 1.81(1.44,2.28)  Adjusted HR- 1.82(1.28,2.59)  Adjusted for parity, gestational hypertension, preterm delivery, size of infants at birth, comorbid conditions, not living with partner at the time of delivery, history of psychiatric illness, history of psychiatric illness in partner, deprivation index level, and ethnocultural background. | Low to moderate risk of bias  Selection bias  Participation rates: 2  Representativeness: 2  Measurement bias  GDM: 1  Mental disorder: 2  Confounding: 2 |
| **Postnatal depression (GDM as exposure, mental disorder as outcome)** | | | | | | | | |
| Bernstein et al, 2017 | USA | Retrospective cohort, N= 12,622 women  GDM- 12,622 | One inpatient or two outpatient ICD-9 codes: 648.8x. | ICD-9 codes (unknown what the codes are) for depression up to one year postpartum. | Live births in OptumLabs Data Warehouse from 31/01/06 -30/09/12; women with GDM diagnosis and first GDM-affected pregnancy taken as the index pregnancy. | Women with non-continuous enrolment, no claims record of a GDM diagnosis, pre-existing diabetes, fetal demise, stillbirths, women without validated demographic characteristics or American Hospital Association Survey data. | Incident cases of postpartum depression- 1.2% of those with GDM (95% CI 1.0-1.4). | Low to moderate risk of bias  Selection bias  Participation rates: 2  Representativeness: 1  Measurement bias  GDM: 1  Mental disorder: 2  Confounding: 1 |
| Mak et al, 2018 | China | Prospective cohort, N=1449  GDM- 229 | 75g 1 hour and 2 hour OGTT at 24-28 weeks gestation. IADPSG criteria- fasting serum glucose ≥5.1 mmol/L, 1-h serum glucose ≥10.0 mmol/L, or 2-h serum glucose ≥8.5 mmol/L. | Chinese version of EPDS at 1 month postpartum and 3 months postpartum. | May-Aug 2015 recruited at four maternity hospitals in Chengdu during routine antenatal care. Age 18–40 years, singleton pregnancy. | Without infertility treatment and absence of any severe chronic or infectious diseases. Excluded women with pre-gestational DM from analysis. | 1 month postpartum EPDS  GDM (mean (SD): 4.3 (3.6)  No GDM: 3.6 (3.5)  p=0.02  3 months postpartum EPDS  GDM (mean (SD): 2.1 (2.3)  No GDM: 1.5 (2.0)  p=<0.001 | Low to moderate risk of bias  Selection bias  Participation rates: 2  Representativeness: 1  Measurement bias  GDM: 2  Mental disorder: 1  Confounding: 1 |
| Meltzer-Brody et al, 2017 | Denmark | Retrospective cohort (population level), N= 392, 458 women  GDM- Unknown | GDM ICD-10 code O24. | Inpatient and outpatient psychiatric diagnoses 0–12 months postpartum (diagnoses given in psychiatric not primary care). All diagnoses of  mental and behavioral disorders (ICD-10 F-chapter)  excluding organic disorders, substance abuse and mental  retardation (ICD-10: F00–F19 and F70–F79). Grouped by: postpartum depression (F32–F33 excluding F32.3), postpartum psychosis  (F20, F23, F25, F28–F31 and F32.3) and reactions to severe stress  (F43). | All women born in Denmark on 01/01/55 or later, who gave birth to a singleton, live-born  child between 01/01/95-30/06/12. First time live births. | Women who  died or migrated before the study start and restricted  the cohort to women without previous history  of psychiatric disorders. | GDM associated with increased risk of all types of postpartum psychiatric disorders IRR 1.28 (1.02,1.62). Not associated with postpartum depression specifically IRR 1.06. | Low to moderate risk of bias.  Selection bias  Participation rates: 2  Representativeness: 1  Measurement bias  GDM: 1  Mental disorder: 2  Confounding: 1 |
| Pace et al, 2018 | Canada | Retrospective cohort, N=58,400 mothers  GDM-29,200 | ICD-9: 6480 or 6488, ICD-10: O24.8. | ICD-9 and 10 codes: ≥2 outpatient visit diagnoses for depression  within a 2 year time frame or one hospitalisation discharge  diagnosis or hospital admission for self-harm or suicide recorded as a cause of death. | Women aged 20–44 years who, between April 1, 1990,  and December 31, 2007, 1) had a singleton live birth and  2) had ≥2 outpatient physician billing diagnoses for GDM  within 6 months  of delivery and/or a postdelivery hospitalisation discharge  GDM diagnosis. | Pre-gestational DM or who had a history of psychiatric illness (mood and psychotic disorders) in the 1 year prior to 24 weeks gestation. | Delivery to 1 year postpartum:  Unadjusted HR for depression in GDM pregnancy- 1.10(0.92,1.32)  Adjusted HR- 1.05(0.85,1.30)  Adjusted for parity, gestational hypertension, preterm delivery, size of infants at birth, comorbid conditions, not living with partner at the time of delivery, history of psychiatric illness, history of psychiatric illness in partner, deprivation index level, and ethnocultural background. | Low to moderate risk of bias  Selection bias  Participation rates: 2  Representativeness: 2  Measurement bias  GDM: 1  Mental disorder: 2  Confounding: 2 |
| **Antenatal anxiety following GDM diagnosis (GDM as exposure, mental disorder as outcome)** | | | | | | | | |
| Daniells et al, 2003 | Australia | Prospective cohort, N=100  GDM- 50 | ADIPS criteria- fasting glucose ≥5.5 and/or 2-h glucose is ≥8.0 mmol/l. | STAI (state-trait anxiety inventory) at 36 weeks gestation. | All women attending the Diabetes  Center eligible  if they had GDM, singleton  pregnancy, not previously diagnosed  with GDM, tested after 26  weeks gestation and seen in clinic both within 1 week of diagnosis  and before 32 weeks gestation. All  women had to be able to read and write  English to give informed consent and had  to be willing to follow the study protocol. | Not specified. | Mean (SD)  State anxiety  GDM-  33.7 (10.9)  No GDM-  35.3 (9.1)  p=0.43  Trait anxiety  GDM-  36.0 (9.0)  No GDM-  37.8 (10.4)  p=0.35  Total for each scale is /80. | High risk of bias  Selection bias  Participation rates: 1  Representativeness: 0  Measurement bias  GDM: 2  Mental disorder: 1  Confounding: 0 |
| Rumbold and Crowther, 2002 | Australia | Prospective cohort, N=209 women  GDM- 25 | 75 g OGTT then diagnosis made according to WHO criteria. | STAI (state-trait anxiety inventory) 6 item short form late in third trimester after GDM diagnosis. | Any English-speaking pregnant women  aged ≥18 attending Adelaide Women and Children’s Hospital for antenatal  care who had either been screened or would later  be screened for GDM. | Women with pre-existing diabetes. | Mean (SD)  GDM- 11(4)  No GDM- 11(4) | High risk of bias  Selection bias  Participation rates: 0  Representativeness: 1  Measurement bias  GDM: 2  Mental disorder: 1  Confounding: 1 |
| **Antenatal anxiety (GDM and mental disorder measured at the same time)** | | | | | | | | |
| Byrn and Penckofer, 2015 | USA | Cross-sectional, N=135 women  GDM- 65 | ‘Had medical data to verify GDM status’ but diagnostic criteria not specified. | STAI (state-trait anxiety inventory) at 24-40 weeks gestation. | Women who received  prenatal care at the research sites,  24-40 weeks gestation, spoke and  read English, had medical data to verify GDM diagnosis,  and were older than age 18. | Women <18, women with T1 or T2DM. | Mean (SD)  State anxiety  GDM-  36.98 (12.28)  No GDM-  34.44 (10.93)  Trait anxiety  GDM-  38.22 (12.77)  No GDM-  34.43 (10.08)  Total for each scale is /80. | Low to moderate risk of bias  Selection bias  Participation rates: 1  Representativeness: 1  Measurement bias  GDM: 1  Mental disorder: 1  Confounding: 1 |
| Daniells et al, 2003 | Australia | Prospective cohort, N=100  GDM- 50 | ADIPS criteria- fasting glucose ≥5.5 and/or 2-h glucose is ≥8.0 mmol/l. | STAI (state-trait anxiety inventory) at 30 weeks gestation. | All women attending the Diabetes  Center eligible  if they had GDM, singleton  pregnancy, not previously diagnosed  with GDM, tested after 26  weeks gestation and seen in clinic both within 1 week of diagnosis  and before 32 weeks gestation. All  women had to be able to read and write  English to give informed consent and had  to be willing to follow the study protocol. | Not specified. | Mean (SD)  State anxiety  GDM-  40.6 (13.3)  No GDM-  34.2 (9.9)  p=0.007  Trait anxiety  GDM-  39.5 (10.3)  No GDM-  38.3 (10.2)  p=0.58  Total for each scale is /80. | High risk of bias  Selection bias  Participation rates: 1  Representativeness: 0  Measurement bias  GDM: 2  Mental disorder: 1  Confounding: 0 |
| Fiskin et al, 2018 | Turkey | RCT baseline,  N=60 women  GDM- 60 | Unknown source of diagnosis or diagnostic criteria. | DASS 42 item measure of anxiety, depression and stress at 24-28 weeks gestation. | Singleton pregnant women with GDM who were on regular polyclinic follow-up of Istanbul University Medical School, Department of Obstetrics and Gynaecology between June 2015–June 2016.  Between 19 and 35, normal weight (BMI <25kg/m²), whose diabetes was regulated by diet. | Pregnant women with pre-existing diabetes and those who were unable to write and understand Turkish. | DASS anxiety score mean (SD)  Experimental arm: 7.2 (4.7)  Control arm: 6.6 (5.3) | High risk of bias  Selection bias  Participation rates: 0  Representativeness: 1  Measurement bias  GDM: 0  Mental disorder: 1  Confounding: 1 |
| Levy-Shiff et al, 2002 | Israel | Prospective cohort, N=153 women (minus 53 with pre-gestational DM)=100 women  GDM- 51 | 1 hour 50g OGTT. If >130 mg/dL, then 3 hour 100g OGTT test performed. ‘Two or more abnormal test values’ but does not specify what they are. | STAI (state-trait anxiety inventory) in second trimester- only state inventory. | Recruited from a high-risk pregnancy clinic in a central hospital. Medical records were screened then women invited to participate. | Not specified. | Mean (SD)  State anxiety  GDM-  1.93 (0.63)  No GDM-  1.76 (0.65)  p=0.46  Total for is /80. | High risk of bias  Selection bias  Participation rates: 2  Representativeness: 0  Measurement bias  GDM: 1  Mental disorder: 1  Confounding: 0 |
| Miazgowski et al, 2018 | Poland | Intervention study (baseline), N=165 women  GDM- 165 | 75g OGTT- diagnosis ‘according to ADA guidelines’. | STAI (state-trait anxiety inventory) at baseline (during pregnancy). | Women with GDM treated in a  tertiary care centre (Outpatient Clinic for Diabetic Pregnant  Women in Szczecin, Poland). | Cases with overt diabetes,  prior GDM, and multiple pregnancies. | Mean (SD)  State anxiety  42.21 (6.12)  Trait anxiety  36.68 (5.86)  Total for each scale is /80. | High risk of bias  Selection bias  Participation rates: 1  Representativeness: 1  Measurement bias  GDM: 2  Mental disorder: 1  Confounding: 0 |
| **Postnatal anxiety (GDM as exposure, mental disorder as outcome)** | | | | | | | | |
| Daniells et al, 2003 | Australia | Prospective cohort, N=100  GDM- 50 | ADIPS criteria- fasting glucose ≥5.5 and/or 2-h glucose is ≥8.0 mmol/l. | STAI (state-trait anxiety inventory) at 6 weeks postpartum. | All women attending the Diabetes  Center eligible  if they had GDM, singleton  pregnancy, not previously diagnosed  with GDM, tested after 26  weeks gestation and seen in clinic both within 1 week of diagnosis  and before 32 weeks gestation. All  women had to be able to read and write  English to give informed consent and had  to be willing to follow the study protocol. | Not specified. | Mean (SD)  State anxiety  GDM-  31.7 (10.6)  No GDM-  34.1 (10.9)  p=0.28  Trait anxiety  GDM-  34.4 (10.5)  No GDM-  36.7 (9.5)  p=0.24  Total for each scale is /80. | High risk of bias  Selection bias  Participation rates: 1  Representativeness: 0  Measurement bias  GDM: 2  Mental disorder: 1  Confounding: 0 |
| Meltzer-Brody et al, 2017 | Denmark | Retrospective cohort (population level), N= 392, 458 women  GDM- Unknown | GDM ICD-10 code O24. | Inpatient and outpatient psychiatric diagnoses 0–12 months postpartum (diagnoses given in psychiatric not primary care). All diagnoses of  mental and behavioral disorders (ICD-10 F-chapter)  excluding organic disorders, substance abuse and mental  retardation (ICD-10: F00–F19 and F70–F79). Grouped by: postpartum depression (F32–F33 excluding F32.3), postpartum psychosis  (F20, F23, F25, F28–F31 and F32.3) and reactions to severe stress  (F43). | All women born in Denmark on 01/01/55 or later, who gave birth to a singleton, live-born  child between 01/01/95-30/06/12. First time live births. | Women who  died or migrated before the study start and restricted  the cohort to women without previous history  of psychiatric disorders. | GDM associated with increased risk of all types of postpartum psychiatric disorders IRR 1.28 (1.02,1.62). Also associated with postpartum reactions to severe stress (F43) (IRR 1.42, 95% CI 1.03,1.97). | Low to moderate risk of bias.  Selection bias  Participation rates: 2  Representativeness: 1  Measurement bias  GDM: 1  Mental disorder: 2  Confounding: 1 |

**Figure S1 Forest plots showing the impact of sensitivity analyses using a leave one out approach based on sample size**

**Forest plot showing pooled odds ratios for antenatal depressive symptoms at the time of GDM diagnosis in women with GDM versus those without GDM using cumulative meta-analysis for a leave one out approach based on sample size**

**Forest plot showing pooled odds ratios for antenatal depressive symptoms in women after a diagnosis of GDM versus those without a diagnosis of GDM using cumulative meta-analysis for a leave one out approach based on sample size**

**Forest plot showing pooled odds ratios for postnatal depressive symptoms in women with GDM versus those without GDM** **using cumulative meta-analysis for a leave one out approach based on sample size**
